# Microbiome Influences Prenatal and Adult Microglia in a Sex-Specific Manner

**DOI:** 10.1016/j.cell.2017.11.042

**Published:** 2018-01-25

**Authors:** Morgane Sonia Thion, Donovan Low, Aymeric Silvin, Jinmiao Chen, Pauline Grisel, Jonas Schulte-Schrepping, Ronnie Blecher, Thomas Ulas, Paola Squarzoni, Guillaume Hoeffel, Fanny Coulpier, Eleni Siopi, Friederike Sophie David, Claus Scholz, Foo Shihui, Josephine Lum, Arlaine Anne Amoyo, Anis Larbi, Michael Poidinger, Anne Buttgereit, Pierre-Marie Lledo, Melanie Greter, Jerry Kok Yen Chan, Ido Amit, Marc Beyer, Joachim Ludwig Schultze, Andreas Schlitzer, Sven Pettersson, Florent Ginhoux, Sonia Garel

**Affiliations:** 1 Institut de Biologie de l’Ecole normale supérieure (IBENS), Ecole Normale Supérieure, CNRS, INSERM, PSL Research University, 75005 Paris, France; 2 Singapore Immunology Network (SIgN), Agency for Science, Technology and Research (A^∗^STAR), Singapore 138648, Singapore; 3 Genomics and Immunoregulation, Life and Medical Sciences (LIMES) Institute, University of Bonn, 53115 Bonn, Germany; 4 Department of Immunology, Weizmann Institute of Science, 76100 Rehovot, Israel; 5 Aix-Marseille Université, CNRS, INSERM, Centre d’Immunologie de Marseille-Luminy (CIML), 13288 Marseille, France; 6 Institut Pasteur, Unité Perception et Mémoire, CNRS, UMR 3571, F-75015 Paris, France; 7 National Cancer Centre, Singapore 169610, Singapore; 8 Institute of Experimental Immunology, University of Zurich, 8057 Zurich, Switzerland; 9 Department of Reproductive Medicine, KK Women’s and Children’s Hospital, Singapore 229899, Singapore; 10 KK Research Centre, KK Women’s and Children’s Hospital, 100 Bukit Timah Road, Singapore 229899, Singapore; 11 Molecular Immunology in Neurodegeneration, German Center for Neurodegenerative Diseases (DZNE), 53127 Bonn, Germany; 12 Platform of Single Cell Genomics and Epigenomics at the German Center for Neurodegenerative Diseases and the University of Bonn, 53175 Bonn, Germany; 13 Myeloid Cell Biology, LIMES-Institute, University of Bonn, 53115 Bonn, Germany; 14 Lee Kong Chian School of Medicine and School of Biological Sciences, Nanyang Technological University, Singapore 639798, Singapore; 15 Department of Microbiology, Tumor and Cell Biology, Karolinska Institute, Stockholm 17165, Sweden

**Keywords:** microglia, sex, microbiome, germ-free, prenatal, antibiotics, embryogenesis, neurodevelopmental disorders, neuroinflammation, CXCR4

## Abstract

Microglia are embryonically seeded macrophages that contribute to brain development, homeostasis, and pathologies. It is thus essential to decipher how microglial properties are temporally regulated by intrinsic and extrinsic factors, such as sexual identity and the microbiome. Here, we found that microglia undergo differentiation phases, discernable by transcriptomic signatures and chromatin accessibility landscapes, which can diverge in adult males and females. Remarkably, the absence of microbiome in germ-free mice had a time and sexually dimorphic impact both prenatally and postnatally: microglia were more profoundly perturbed in male embryos and female adults. Antibiotic treatment of adult mice triggered sexually biased microglial responses revealing both acute and long-term effects of microbiota depletion. Finally, human fetal microglia exhibited significant overlap with the murine transcriptomic signature. Our study shows that microglia respond to environmental challenges in a sex- and time-dependent manner from prenatal stages, with major implications for our understanding of microglial contributions to health and disease.

## Highlights

• Microglia undergo sequential phases of differentiation during development
• The maternal microbiome influences microglial properties during prenatal stages
• The absence of the microbiome has a sex- and time-specific impact on microglia
• Microbiome depletions have acute and long-term effects on microglial properties

## Graphical Abstract

**Figure:**
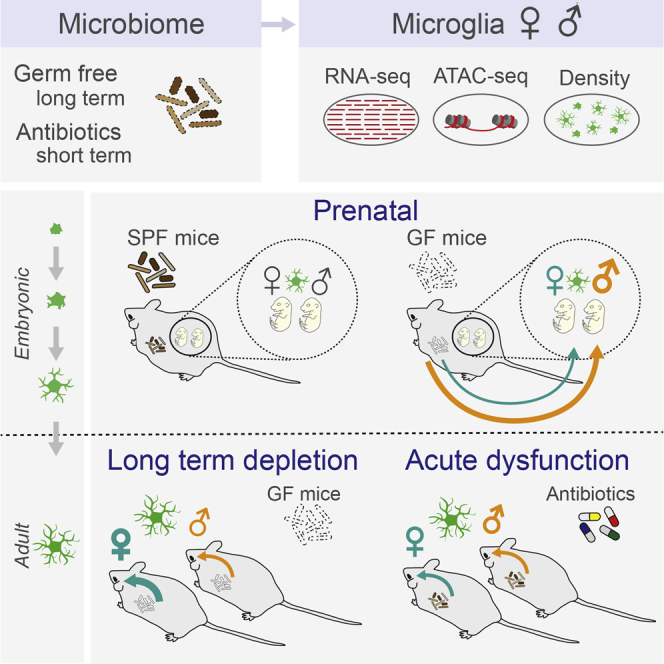

## Teaser

Microglia respond to environmental challenges, such as signals from the gut microbiome, in a sex- and time-dependent manner.

## Introduction

Microglia, the resident macrophages of the CNS, constitute the first line of defense against injury and infections. They originate from yolk-sac macrophages (YSM), enter the brain when the first neurons are generated (around embryonic day [E] 9.5 in mice) (Casano and Peri, 2015, Ginhoux and Prinz, 2015, Prinz et al., 2017), expand, and self-renew in adulthood (Tay et al., 2017a, Thion and Garel, 2017). Alongside their immune roles, recent studies have shown that both fetal and adult microglia also contribute to a variety of processes including brain development, homeostasis, and function. At the cellular or circuit level, microglia regulate synaptic transmission, synaptic pruning and formation, cell death and survival, as well as embryonic wiring (Hong et al., 2016, Ransohoff and El Khoury, 2015, Reemst et al., 2016, Schafer and Stevens, 2015, Tay et al., 2017b, Thion and Garel, 2017, Volk, 2017, Wolf et al., 2017). Consistent with their diverse roles, microglia have been linked to the initiation or progression of several developmental and neurodegenerative diseases, including autism spectrum disorders (ASD), schizophrenia, Alzheimer’s disease, Parkinson’s disease (PD), several auto-immune diseases, and multiple sclerosis (MS) (Colonna and Butovsky, 2017, Derecki et al., 2014, Hong et al., 2016, Shemer and Jung, 2015). To perform both immune and neuronal functions, microglia continuously probe their environment (Gosselin et al., 2014, Gosselin et al., 2017, Lavin et al., 2014, Matcovitch-Natan et al., 2016) via an array of receptors and signaling molecules collectively called the sensome (Hickman et al., 2013).

In recent years, the microbiome has emerged as a key regulator of brain circuitry, neuro-physiology, and behavior (Braniste et al., 2014, Diaz Heijtz et al., 2011, Sharon et al., 2016). As such, the absence of gut microbiota in germ-free mice (GF) or its dysbiosis constitutes an aggravating factor in mouse models of ASD and PD (Hsiao et al., 2013, Sampson et al., 2016). Remarkably, the lack of microbiome perturbs microglial properties via the release of short-chain fatty acids (Erny et al., 2015, Matcovitch-Natan et al., 2016). Thus, microglia that lie at the interface between environmental signals and brain circuitry throughout embryonic and adult life are prime candidates for contributing to these effects.

In addition to extrinsic signals, intrinsic factors such as sexual identity may play important roles in microglial function. Rodent microglia exhibit sexually dimorphic properties in pain perception, contribute to brain masculinization, and show differences in brain colonization in males and females (Lenz and McCarthy, 2015, Mapplebeck et al., 2016, Schwarz et al., 2012). Furthermore, it was recently reported that microglia show transcriptomic differences in females and males along postnatal development (Hanamsagar et al., 2017). Given that some disorders show marked sexual bias, with, for instance, ASD affecting more males and auto-immune diseases more females (McCarthy and Wright, 2017, Nelson and Lenz, 2017), it will be essential to dissect how sexual identity affects microglial differentiation or function.

Here, we asked how the microbiome and intrinsic properties of males and females contribute to the differentiation and maturation of microglia. We performed developmental transcriptomic and chromatin accessibility analyses on microglia purified from male and female mice under either specific-pathogen free (SPF) or GF conditions at different stages of development and found that microglia undergo distinct phases of differentiation, start expressing sensome genes *in utero*, and acquire sexually dimorphic transcriptomic profiles postnatally. Intriguingly, microglia from male and female mice responded differently to the permanent lack of microbiome: GF conditions most severely affected embryonic microglia in males, whereas in females the most marked perturbations were seen in adults. Antibiotic treatment of adult mice revealed that microbiome depletion has both short- and long-term impact on microglia with distinct sexually biased components. Finally, in human fetal microglia, we uncovered significant overlap with the murine microglial transcriptome and a parallel lack of microglial sexual dimorphism in mid-gestation. Taken together, our work reveals that the maternal microbiome influences the maturation of embryonic microglia. Furthermore, it shows a remarkable sexually dimorphic response of microglia to environmental perturbations, which has major implications for our comprehension of the roles of these cells in physiological and pathological conditions.

## Results

### Microglia Acquire Sensome Gene Expression during Developmental Phases

**Figure 1:**
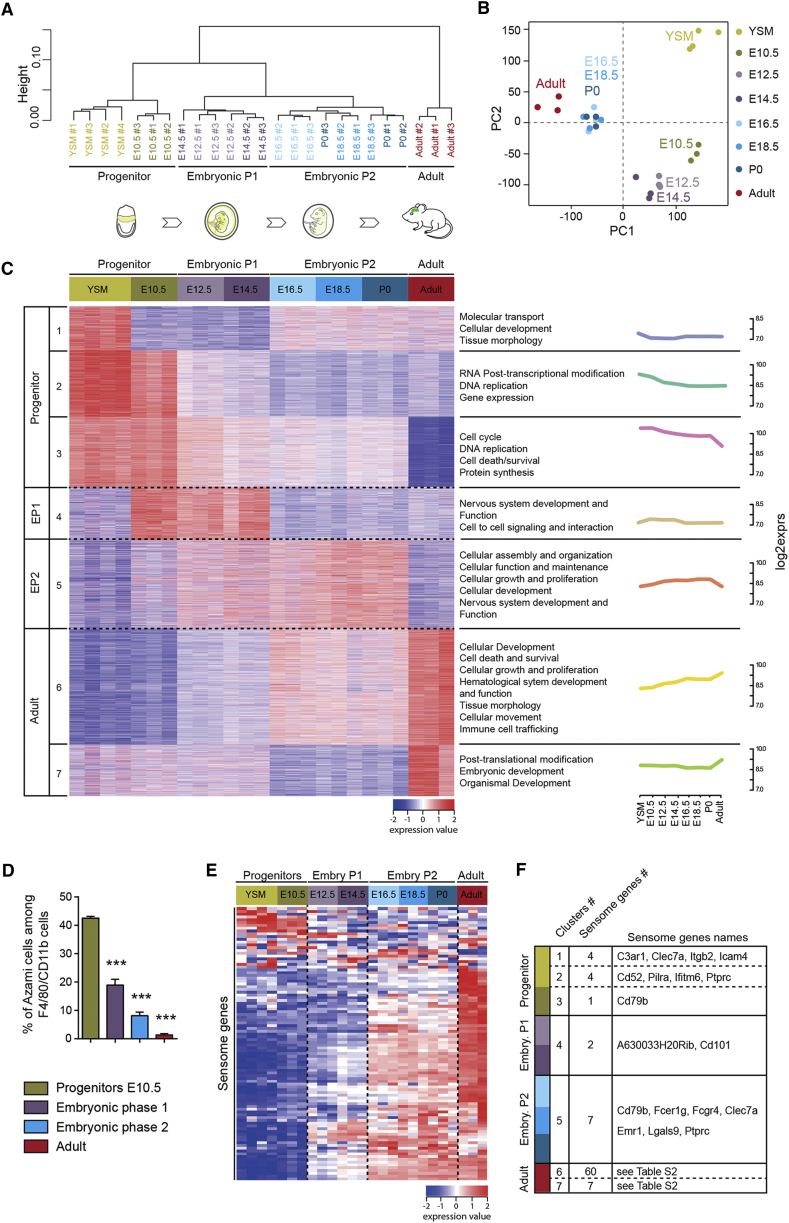
Microglia Undergo Distinct Developmental Phases (A and B) Dendrogram (A) and PCA (B) on transcriptomes of murine YS progenitors and microglia at different developmental stages. n = 3–4 replicates per stage, with each replicate obtained by pooling microglia sorted from several female and male brains. PC, principal component. (C) Heatmap of the DEGs with clusters (left), associated signaling pathways (right), and corresponding expression plots. Each row is a biological replicate. (D) Percentages of Azami green^+^ cells (S/G2/M cell-cycle phases) among F4/80/CD11b-positive cells from brains of FUCCI mice. Data are represented as means ± SEM; n = 3–5 per stage; one-way ANOVA with Tukey post hoc test was used to assess differences; ^∗∗∗^p < 0.001. (E) Heatmap of the expression level of microglia sensome genes. (F) Microglial sensome gene expression in the different developmental clusters. Embryo P, embryonic phase. See also Figures 2, S1, and Table S1.

**Figure S1:**
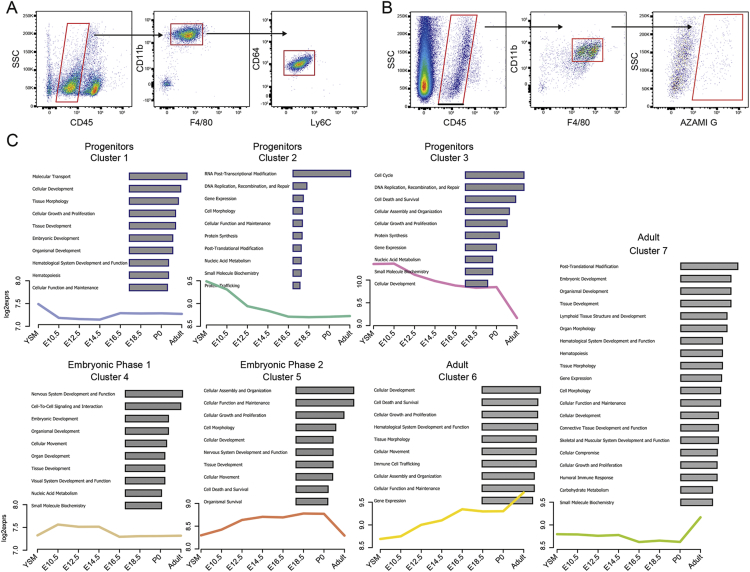
Microglial Changes during Development, Related to Figure 1 (A) Gating strategy for flow cytometry purification of CD45^low^, CD11b^+^, F4/80^+^, CD64^+^, Ly6C^-^ yolk-sac progenitors and microglia. (B) Gating strategy of flow cytometric analysis of cells from FUCCI mice showing CD45^low^, CD11b^+^, F4/80^+^, and Azami green^+^ cells. (C) The seven clusters characterizing the different developmental phases of microglia. The plots show the expression of the corresponding genes that are associated with these functions during development. Clusters 1, 2 and 3 are related to the progenitor phase, cluster 4 to embryonic phase 1, cluster 5 to embryonic phase 2 and clusters 6 and 7 to the adult stage. See also Table S1.

To investigate how microglia differentiate, we first performed microarrays to examine gene expression profiles of fluorescence-activated cell sorting (FACS)-purified YS progenitors and microglia, at five embryonic stages, on the day of birth (P0), and in adults (P60), by pooling microglia from males and females together (Figures 1A–1C and S1A–S1C). Using unsupervised hierarchical clustering and principal component analysis (PCA), we identified distinct phases of differentiation (Figures 1A and 1B): (1) progenitor phase (YSM and E10.5), (2) embryonic phase 1 (E12.5 to E14.5), (3) embryonic phase 2 (E16.5 to P0), and (4) adult stage, consistent with previous studies (Matcovitch-Natan et al., 2016). To examine biological differences between phases, we performed gene ontology (GO) analyses on significantly differentially expressed genes (DEGs) (Figures 1C and S1C; Table S1). The progenitor phase was characterized by enriched expression of 4,582 genes (clusters 1 to 3) involved in cell cycle, proliferation, and DNA replication, including *Klf9* and *E2f6* (Figures 1C and S1C), consistent with anatomical studies (Swinnen et al., 2013). To assess proliferation, we used FUCCI mice, in which cell-cycle phases can be visualized by expression of fluorescent protein reporters (Sakaue-Sawano et al., 2008), and confirmed that CD45^+^CD11b^+^ cells, likely representing microglial precursors (Ginhoux et al., 2010), displayed the highest proliferative rate during the progenitor phase (Figures 1D and S1B). Embryonic phases 1 (1,299 DEGs; cluster 4) and 2 (2,116 DEGs; cluster 5) were characterized by high expression of genes linked with nervous system development and function, cellular assembly and organization, cell-to-cell signaling, and cellular movement, including *PlexinA2*, *Cxcr4*, and *Igf1* (Figures 1C and S1C) (Marín, 2013, Ueno et al., 2013). Finally, the adult stage was characterized by differential expression of 3,508 genes (clusters 6 and 7) involved in cellular development and immune activation, including *Ccr5*, *Mafb*, and *Jun* (Figures 1C and S1C) (Matcovitch-Natan et al., 2016). Thus, our analysis indicates that microglia exhibit the potential for specific functions at distinct stages of brain development. One key function of microglia is to respond to their environment through the expression of the sensome genes, which were first described in adult microglia (Hickman et al., 2013). We found that 9 sensome genes were specifically highly expressed in the progenitor phase and 9 others in embryonic phases, and the majority of the sensome genes showed highest levels of transcripts in adults (Figures 1E and 1F; Table S1). Thus, microglia begin to express sensome genes *in utero*, raising the possibility that they could respond to local or systemic changes during embryogenesis.

### Developmentally Regulated Networks and CXCR4 Function in Brain Colonization

**Figure 2:**
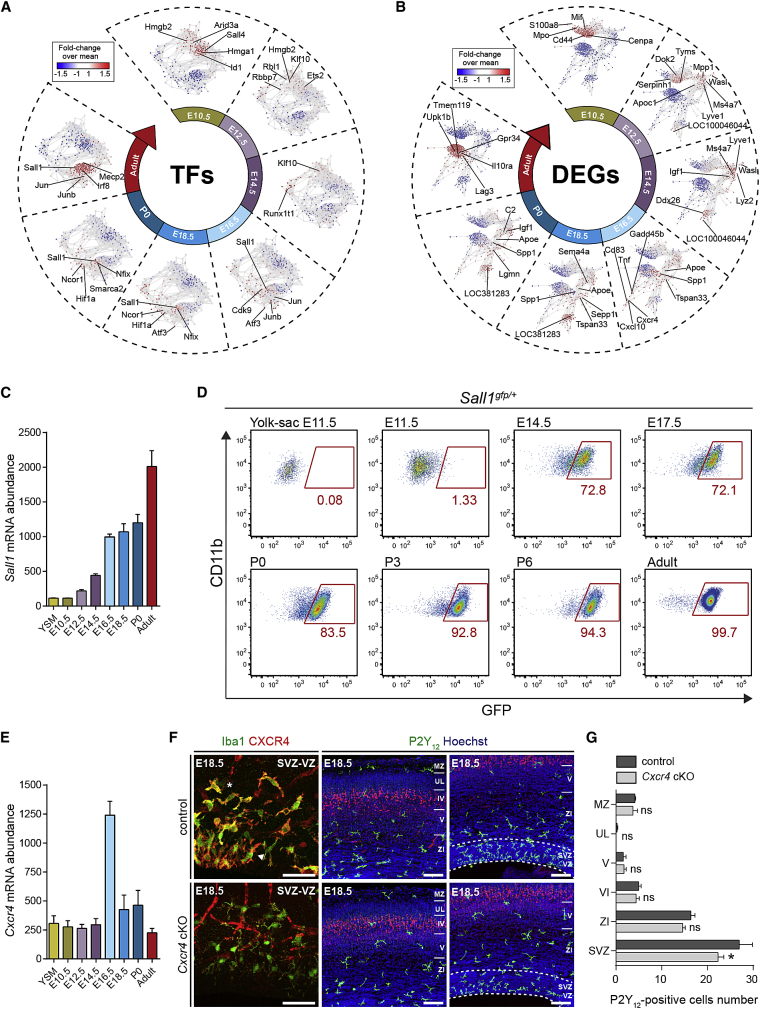
Regulation of Microglial Gene Expression during Development and the Impact of CXCR4 on Microglial Brain Colonization (A and B) Visualization of co-expression networks analysis (CENA) based on the expression of 431 transcription factors (TFs) (A) and on the expression of DEGs (B) (n = 3–4 biological replicates per stage; −1.5 < fold-change < 1.5 and false discovery rate [FDR]-corrected p value < 0.05). Expression differences relative to the overall mean are shown by node color on the CENA network. (C) *Sall1* mRNA levels abundance from microarray dataset. (D) Flow cytometry analysis of GFP^+^ cells in *Sall1*^*gfp/+*^ mice within microglia (CD45^+^Ly6C^−^Ly6G^−^F4/80^+^CD11b^+^). n = 6–11 per stage. (E) *Cxcr4* mRNA levels abundance from microarray dataset. (F) E18.5 coronal sections of the somatosensory neocortex showing Iba1 expression, P2Y12 and CTIP2 immunostainings in controls, and CXCR4 downregulation in *Cxcr4* cKO mice. Scale bars, 50 μm (left) or 100 μm (right). (G) Number of P2Y12-positive cells in the somatosensory cortex of control and *cxcr4* cKO mice. n = 3–4 mice per condition. Data are represented as means ± SEM; two-way ANOVA with Sidak post hoc test was performed to assess differences at each stage. ^∗^p < 0.05. See also Figures 1, S1, and Table S1.

To understand how microglia evolves during differentiation, we applied co-expression network analyses (CENA) (Ulas et al., 2017), which highlight gene clusters strongly expressed in each phase as well as their temporal evolution, focusing either on transcription factors (TFs) or all DEGs (Figures 2A and 2B; Table S1). Genes encoding TFs, including *Sall1* or *Irf8* that have been linked to microglial differentiation (Buttgereit et al., 2016, Kierdorf et al., 2013, Masuda et al., 2012), were dynamically regulated across phases, reflecting the progression of microglial maturation (Figures 2A and 2C). Using *Sall1*^*gfp/+*^ mice (Takasato et al., 2004), we confirmed this temporal progression: few cells expressed *Sall1* at E11.5, 72.8% of microglia were GFP^+^ at E14.5, and almost all the cells were labeled in adults (Figure 2D).

To assess whether the identified DEGs might regulate early microglial activity, we focused on genes specifically expressed during embryonic phases, such as *Cxcr4.* This gene encodes a chemokine receptor expressed by microglia and was proposed to regulate microglial colonization in response to focal expression of its ligand, CXCL12 (Arnò et al., 2014). The abundance of *Cxcr4* transcripts peaked at E16.5 (Figure 2E), and protein expression was confirmed at E18.5 in the neocortex (Figure 2F). To investigate the role of this receptor, we generated mice in which *Cxcr4* could be conditionally knocked out (*Cxcr4* cKO) using tamoxifen-inducible cre under the control of c*x3cr1* (*Cx3cr1*^*creERT2/+*^;*Cxcr4*^*flox/flox*^ mice), which is expressed by microglia (Yona et al., 2013). Conditional inactivation of microglial *Cxcr4* was confirmed by CXCR4 immunostaining (Figure 2F). Using specific markers to delineate cortical layers, we observed a significant and region-specific decrease in the number of microglia in the neocortex SVZ/VZ of cKO at E18.5, 2 days after the peak of *Cxcr4* expression (Figures 2E and 2G). These results indicate a cell-autonomous role of CXCR4 in the embryonic distribution of microglia and highlights the validity of our approach to identify candidate regulators of stage-dependent microglial activity.

### Adult Microglia Acquire Sexually Dimorphic Transcriptomic Signatures

**Figure S2:**
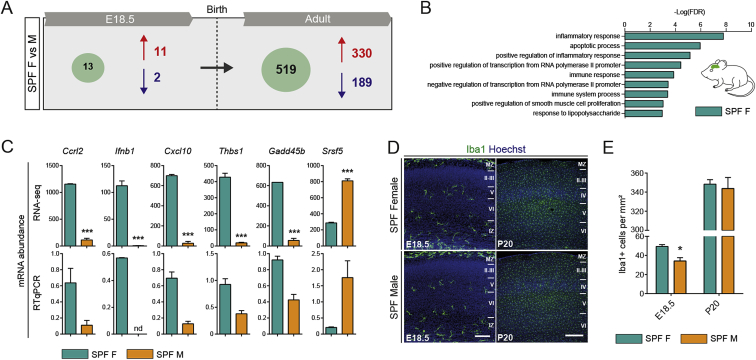
Microglia Progressively Acquire a Sex-Linked Transcriptomic Signature, Related to Figure 3 (A) Number of genes showing at least a 1.5-fold difference in expression level between microglia from SPF female and SPF male mice at E18.5 or in adults. n = 2-3 per stage and condition. (B) Signaling pathways analysis of the DEGs showing at least 1.5-fold greater expression in microglia from SPF females compared to SPF males at E18.5. FDR, False Discovery Rate. (C) RNA-seq and RTqPCR validation of expression data for six DEGs showing at least 1.5-fold difference in expression level in microglia from adult SPF females and SPF males. n = 2-3 per condition. (D) Coronal sections of the somatosensory cortex from E18.5 and adult mice showing Iba1 expressing microglia in SPF female and SPF male (representative of 6 samples per condition). Scale bar E18.5 = 100 μm; scale bar P20 = 300 μm. (E) Microglial density in the somatosensory cortex of male and female mice at E18.5 and in adults. Two-sided unpaired Mann-Whitney test was performed to assess differences at each stage. n = 6 per condition. For all panels, data are represented as means ± SEM. ^∗∗∗^ p < 0.001, ^∗^ p < 0.05, nd, not determined. Similar to Figures 4D and 4E. See also Tables S2 and S3.

To investigate whether sexual identity influences microglial development, we performed RNA-sequencing (RNA-seq) on FACS-purified microglia from E18.5, shortly after the initiation of production of sex hormones (Nelson and Lenz, 2017), as well as from adult brains of female and male SPF mice (Figure S2). At E18.5, microglia purified from female and male brains displayed low numbers of DEGs mostly present on the X and Y chromosomes (Figure S2A; Table S2). This limited embryonic transcriptomic sexual dimorphism increased in adult females and males (Figures S2A and S2E), consistent with recent studies (Hanamsagar et al., 2017): female microglia displayed higher expression of genes associated with inflammatory response, apoptotic process, and response to lipopolysaccharide (LPS) (Figure S2B; Table S2), revealed by GO analyses using the Database for Annotation, Visualization and Integrated Discovery (DAVID). Thus, microglia appeared to be in a more immune-activated state in females, in line with previous studies showing stronger innate and adaptive immune responses in females (Klein and Flanagan, 2016). Among the adult DEGs, expression of 9 genes described as interferon-stimulated genes (*S100a8*, *S100a9*, *Ifit1*, *Ifit2*, *Cxcl10*, *Ccl2*, *Irf1*, *Ccnd3*, and *Gbp5*) was higher in female SPF mice, and the expression of sensome genes, such as *Gpr34* and *Ccrl2*, also differed between males and females (Table S2). We confirmed the differential expression of several DEGs using real-time qPCR (qRT-PCR, n = 12 DEGs) (Figure S2C; Table S3).

Furthermore, we examined the distribution of microglia in males and females that have been reported to exhibit sex-specific differences in microglial density (Lenz and McCarthy, 2015). As illustrated in the neocortex, while there was a small transient increase in microglial density at E18.5 in females as compared to males, the colonization was largely similar in both sexes (Figures S2D and S2E). Thus, adult microglia show some sex-specific divergence in their transcriptomic signatures although with limited differences in their colonization patterns.

### Impact of Microbiota on Microglial Transcriptome Varies According to Stage and Sex

**Figure 3:**
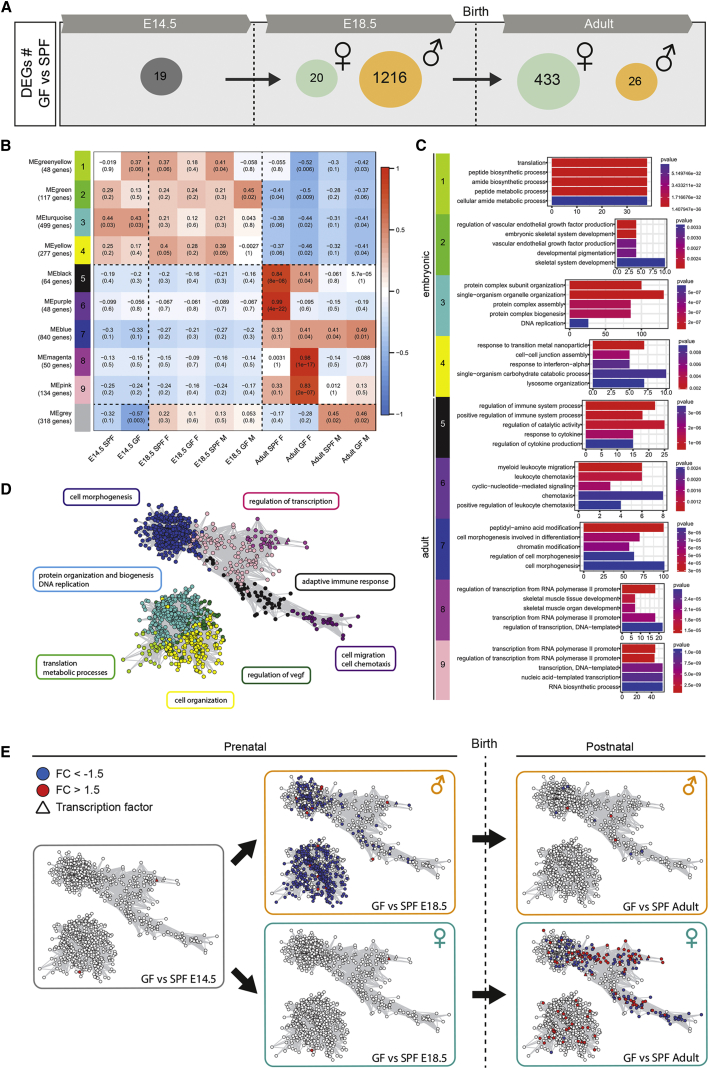
Absence of Microbiota Has a Sex- and Time-Specific Impact on Microglial Transcriptomic Profiles (A) Number of DEGs showing a 1.5-fold difference in expression level between microglia from SPF and GF mice at E18.5 and in adults. n = 2–3 replicates per condition and stage, with embryonic replicate obtained by pooling microglia from 3–7 brains. (B) Module-trait correlation analysis. Each row represents a module eigengene (ME) and each column a trait. Corresponding correlation (top) and p values (bottom) are indicated for each cell. (C) GO terms associated with each module, ranked by p value with top 5 processes listed. (D) Graphic representation (Cytoscape) of the co-expression network is based on all genes in weighted gene co-expression network analysis (WGCNA) having a topological overlap with at least one other gene of at least 0.3. Clusters with fewer than 5 nodes were excluded. Nodes are colored according to module membership. (E) Differential gene expression levels of microglia from GF and SPF brains within the gene co-expression network. Blue and red nodes represent DEGs with a fold-change (FC) <1.5 or >1.5, respectively. See also Figures S2, S3, and Table S2.

**Figure S3:**
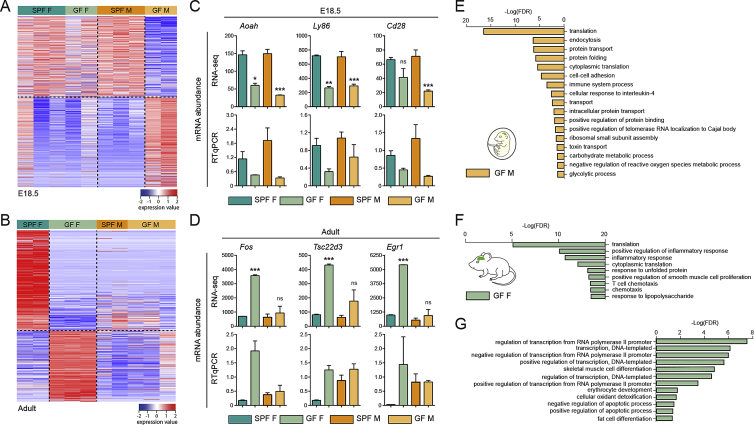
Absence of Microbiota Has Sex- and Stage-Specific Impacts on the Microglial Transcriptome, Related to Figure 3 (A) Heatmap of differentially-expressed genes (DEGs) in microglia between E18.5 GF male and SPF male mice. SPF female and GF female microglial gene expression is also depicted. Each row represents a biological replicate; n = 2-3 replicates per condition. (B) Heatmap of DEGs in between microglia from adult GF females and SPF females. Gene expression in microglia from SPF males and GF males is also depicted. Each row represents a biological replicate; n = 2-3 replicates per condition. (C) RNA-seq and RTqPCR validation of expression data for three DEGs showing a 1.5-fold difference in expression between microglia from E18.5 GF males versus SPF males, or E18.5 GF females versus SPF females. n = 2-3 replicates per condition. (D) RNA-seq and RTqPCR validation of expression data for three DEGs showing a 1.5-fold greater level of expression in microglia from adult GF females compared to SPF females. n = 2-3 replicates per condition. (E) Signaling pathways analysis of the DEGs showing at least 1.5-fold lower expression in microglia from GF male compared to SPF male at E18.5. FDR, False Discovery Rate. (F) Signaling pathways analysis of the DEGs showing at least 1.5-fold lower expression level in microglia from adult GF females compared to SPF females. FDR, False Discovery Rate. (G) Signaling pathways analysis of the DEGs showing at least 1.5-fold higher expression level in microglia from adult GF females compared to SPF females. FDR, False Discovery Rate. For all panels, data are represented as means ± SEM; ^∗^p < 0.05, ^∗∗∗^p < 0.001; ns, not significant. See also Tables S2 and S3.

Considering the effect of gut microbiota in adults (Erny et al., 2015, Matcovitch-Natan et al., 2016), we asked whether the maternal microbiome was similarly important for microglial development before birth. We performed RNA-seq on FACS-purified microglia from the brains of E14.5 and E18.5 embryos from GF and SPF dams and from GF and SPF adults (P60) (Figure 3A; Table S2). At E14.5, the effect was subtle with only 19 DEGs between SPF and GF microglia (Figure 3A). These genes included *Ly86* and *Aoah*, which are involved in LPS processing and response (Lu et al., 2008, Nagai et al., 2002) suggesting that the microbiome regulates the early expression of genes controlling its own detection and responses to inflammation. Closer to birth, , the absence of maternal microbiota had a more profound and sex-specific impact on microglial transcriptomic profiles: 1,216 genes were differentially expressed between E18.5 male microglia from SPF and GF embryos, whereas only 20 genes were differentially expressed between female microglia from SPF and GF embryos at the same time point (Figures 3A and S3A). Most of the DEGs in GF males were expressed at a lower level (1,169 versus 47 expressed at a higher level). In contrast, the magnitude of the effect was reversed between the sexes in adults: 433 genes were differentially expressed in microglia from female GF mice compared to female SPF (226 at higher level and 207 at lower level), whereas just 26 genes were deregulated in males (Figures 3A and S3B). Changes in expression levels were validated by real-time qPCR on selected genes (n = 15 DEGs at E18.5 and n = 12 DEGs in adults) (Figures S3C and S3D; data not shown). DAVID analysis indicated that microglial DEGs in male GF embryos were linked to translation and metabolism, whereas microglia from female GF adults showed lower expression of genes associated with the inflammatory response and enhanced expression of genes linked with regulation of transcription (Figures S3E–S3G; Table S2).

To identify the biological networks affected by sexual identity, developmental stage, and the absence of microbiota, we performed weighted gene co-expression network analysis (WGCNA) (Figures 3B and 3E). This approach enabled us to identify modules of genes whose expression was differently regulated in microglia from embryonic and adult stages, females and males, or SPF and GF microglia (Figures 3B and 3D). Four modules were specific for embryonic stages, mainly associated with translation, protein organization and biogenesis, metabolic processes, and cell organization. Five modules characterized adult microglia. These modules contained genes linked to immune responses, cell migration and chemotaxis, cell morphogenesis, and regulation of transcription (Figures 3B and 3D). We next mapped the DEGs between E18.5 SPF versus E18.5 GF and adult SPF versus adult GF microglia. Overlaying these DEGs onto the network topology revealed that during the embryonic stages, the absence of maternal microbiome drastically downregulates the expression of genes that are associated with metabolic processes, cell and protein organization, and adaptive immune response specifically in males, whereas E18.5 GF female microglia did not display a major perturbation of gene expression in the network (Figure 3E, clusters 2, 3, 4, and 7). In adults, GF female microglia exhibited many dysregulated genes linked with cell morphogenesis, regulation of transcription, adaptive immune responses, and cell migration and chemotaxis (Figure 3E), whereas GF male microglia did not show major changes. Thus, there is a striking impact of the absence of maternal microbiota on embryonic male microglial gene expression and of continued GF status on adult female microglia. Collectively, our work reveals that microglia already respond to the absence of the maternal microbiome from prenatal stages and furthermore respond in a stage-specific manner in males and females.

### Microglial Colonization in Germ-Free Mice Is Altered in a Time- and Sex-Specific Way

**Figure S4:**
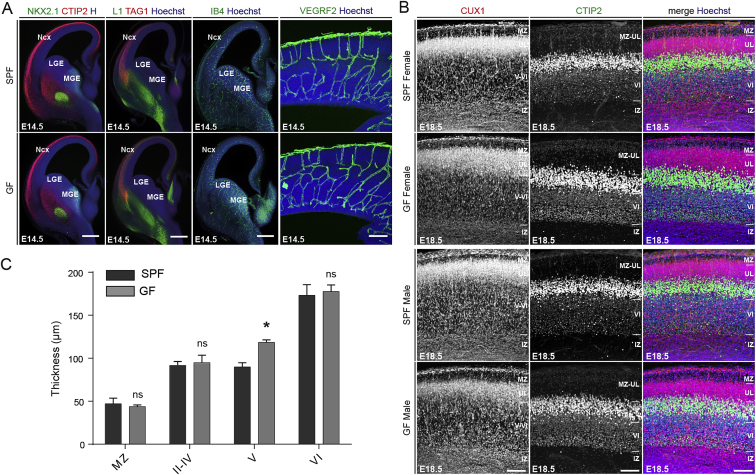
Brain Patterning and Cortical Layering in GF Mice, Related to Figure 4 (A) Coronal sections of brains from SPF and GF mice showing NKX2.1, CTIP2, L1, IB4, VEGFR and TAG1 immunofluorescences at E14.5. Scale bars low magnification = 500 μm; scale bar high magnification = 100 μm. n = 3-4 replicates by conditions. (B) Coronal sections of the somatosensory cortex of brains from SPF and GF male and female mice, showing CUX1-positive layer II-III-IV and CTIP2-positive layer V immunofluorescence at E18.5. Scale bars = 100 μm. (C) Measurement of cortical layer thickness at E18.5 in brains from SPF and GF mice. n = 4 replicates by conditions and sex. Data are represented as means ± SEM; Two-way ANOVA with Sidak post hoc test was performed to assess differences at each stage; ^∗^p < 0.05, ns, not significant.

We next wanted to determine whether GF status changes embryonic microglia colonization and morphology as reported in adults (Erny et al., 2015). We first confirmed by histology and immunostaining that GF embryos do not show gross differences in forebrain patterning, axonal tract development, or blood vessel formation compared to SPF controls (Figure S4A). We further examined cortical layering in the E18.5 somatosensory cortex and while there were no major differences, the thickness of layer V was increased in all GF animals when compared to the SPF controls (Figures S4B and S4C). Thus, GF mice, while presenting some minor deficits, show a relatively preserved brain morphogenesis.

**Figure 4:**
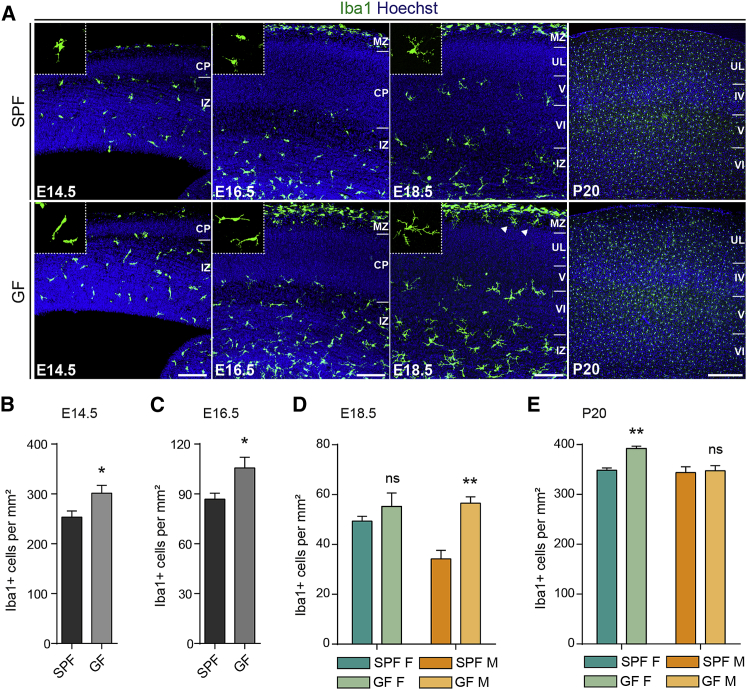
The Absence of Microbiota Has a Sex- and Time-Specific Impact on Microglial Colonization of the Neocortex (A) Coronal sections of the somatosensory neocortex of SPF and GF mice showing Iba1^+^ cells. Scale bars 100 μm for E14.5–E18.5 and 300 μm for adults. (B–E) Density of Iba1^+^ cells in the somatosensory neocortex of SPF and GF mice at (B) E14.5 (n = 7–8), (C) E16.5 (n = 7–10), (D) E18.5 (n = 4–5), and (E) P20 (n = 3–6). Data are represented as means ± SEM. Two-sided unpaired Mann-Whitney test was performed to assess differences at E14.5 and E16.5, and two-way ANOVA with Sidak post hoc test was performed to assess differences at E18.5 and P20. ^∗^p < 0.05, ^∗∗^p < 0.01, ns, not significant. Same SPF samples as in Figure S2. See also Figures S4, S5, and S6.

**Figure S5:**
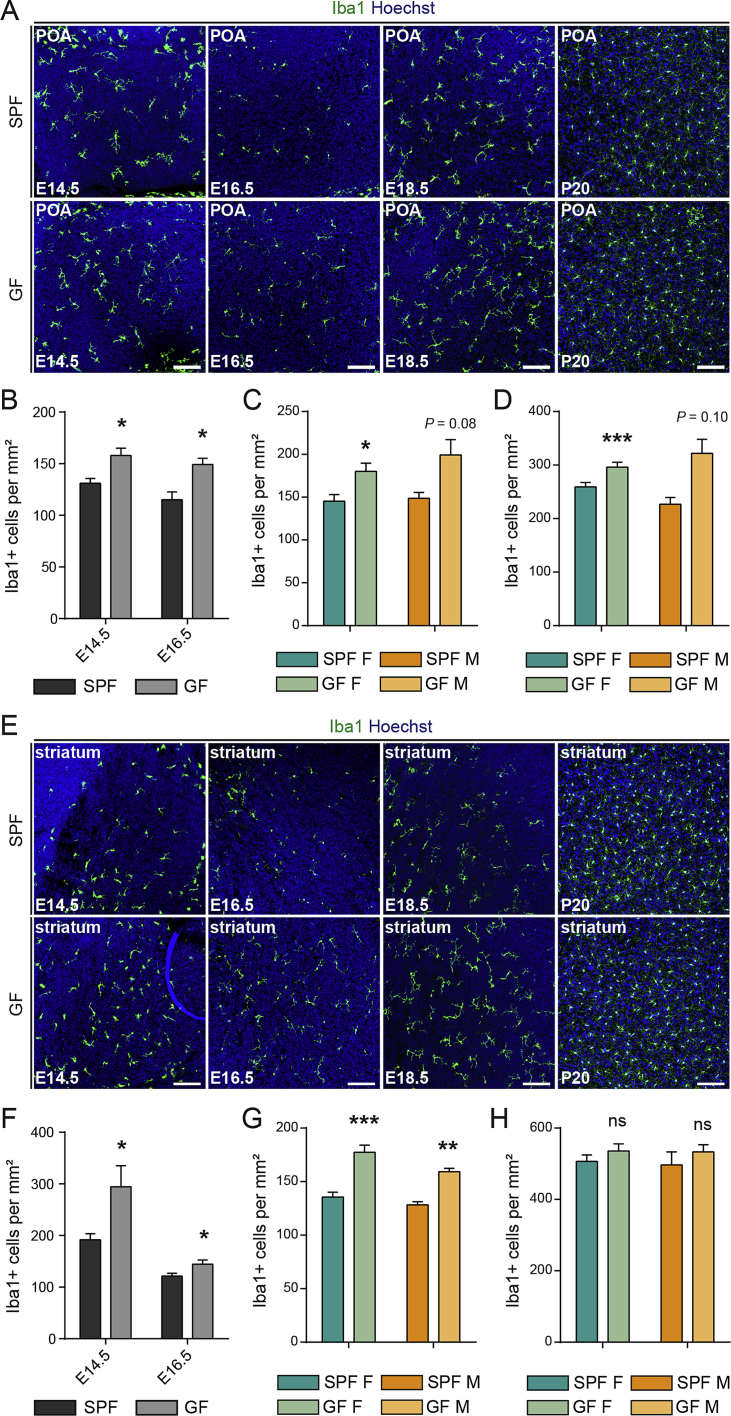
Colonization of Microglia in the Preoptic Area and Striatum of Brains of GF Mice, Related to Figure 4 (A) Coronal sections of the preoptic area (POA) of brains from SPF and GF mice at different stages of development (E14.5, E16.5, E18.5 and P20) showing Iba1 immunohistochemistry. Scale bars = 100 μm. (B) Density of Iba1-positive cells in the POA of brains from SPF and GF mice at E14.5 and E16.5. n = 7-11 per stage and condition. (C) Density of Iba1-positive cells in the POA of brains from female and male mice under SPF or GF conditions at E18.5. n = 4-5 per stage and condition. (D) Density of Iba1-positive cells in the POA of brains from female and male mice under SPF or GF conditions at P20. n = 3-7 per stage and condition. (E) Iba1 labeling of coronal sections of the striatum of brains from SPF and GF mice at different stages of development (E14.5, E16.5, E18.5 and P20). Scale bars = 100 μm. (F) Density of Iba1-positive cells in the striatum of brains from SPF and GF mice at E14.5 and E16.5. n = 7-11 per stage and condition. (G) Density of Iba1-positive cells in the striatum of brains from female and male mice under SPF or GF conditions at E18.5. n = 4-5 per stage and condition. (H) Density of Iba1-positive cells in the striatum of brains from female and male mice under SPF or GF conditions at P20. n = 3-6 per stage and condition. Data are represented as means ± SEM. Two-sided unpaired Mann-Whitney test was performed to assess differences at E14.5 and E16.5 and Two-way ANOVA with Sidak post hoc test was performed to assess differences at E18.5 and P20; ^∗^p < 0.05, ^∗∗^p < 0.01, ^∗∗∗^p < 0.001, ns, not significant.

**Figure S6:**
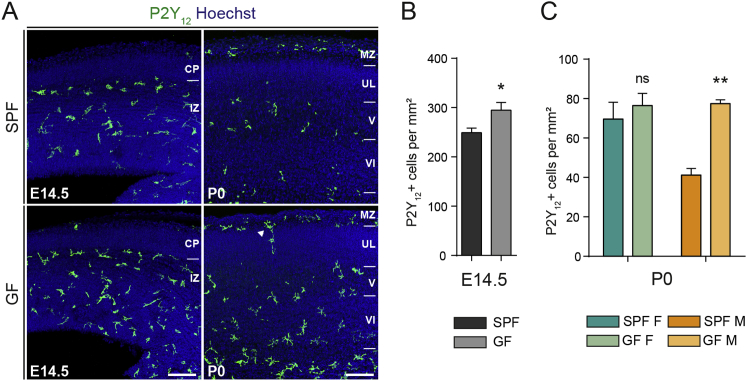
Absence of Microbiota Has a Sex-Specific Impact on Microglial Colonization of the Neocortex, Related to Figure 4 (A) Coronal sections of the somatosensory neocortex of SPF and GF mice at different stages of development (E14.5 and P0) showing P2Y12-positive microglia. Scale bars E14.5-E18.5 = 100 μm. (B) Density of P2Y12-positive cells in the somatosensory neocortex of SPF and GF mice at E14.5. n = 7-8 per condition. (C) Density of P2Y12-positive cells in the cortical plate of the somatosensory neocortex of female and male SPF and GF mice at P0. n = 4 per stage and condition. Data are represented as means ± SEM. Two-sided unpaired Mann-Whitney test was performed to assess differences at E14.5 and Two-way ANOVA with Sidak post hoc test was performed to assess differences at P0; ^∗^p < 0.05, ^∗∗^p < 0.01, ns, not significant.

We next assessed microglial density and morphology using Iba1 staining at E14.5, E16.5, and E18.5, and at postnatal day 20 (P20), in both SPF and GF conditions (Figures 4 and S5). The brains of GF mice exhibited increased densities of embryonic microglia in all three regions examined, namely the somatosensory cortex, the striatum, and the POA at E14.5 and E16.5 (Figures 4A–4C and S5). In addition, embryonic microglia from GF mice displayed excessive ramification, as reported in adults (Erny et al., 2015). Later in development, in the somatosensory cortex, this phenotype was retained and also exhibited a sexual bias, consistent with our transcriptomic analyses: at E18.5, microglial density was significantly increased in the brains of males versus females, whereas females exhibited increased microglial density relative to males postnatally (Figures 4D and 4E). Such sex- and stage-dependent regulation of microglial density appeared also to be region-specific. Indeed, in the POA and striatum, both sexes displayed increased microglial density at E18.5 in GF conditions as compared to SPF controls, and no clear difference in density was observed in the GF adult striatum (Figures S5C and S5G). Because Iba1 staining can also label other brain myeloid cells, such as perivascular macrophages, we confirmed the increase in microglial density using a second, more specific microglial marker, P2Y12 (Mildner et al., 2017) (Figure S6). Taken together, both transcriptomic and colonization analyses support sex- and age-specific perturbations of microglia in GF mice.

### ATAC-Seq Reveals Temporal Changes in Chromatin Accessibility in Germ-Free Mice

**Figure 5:**
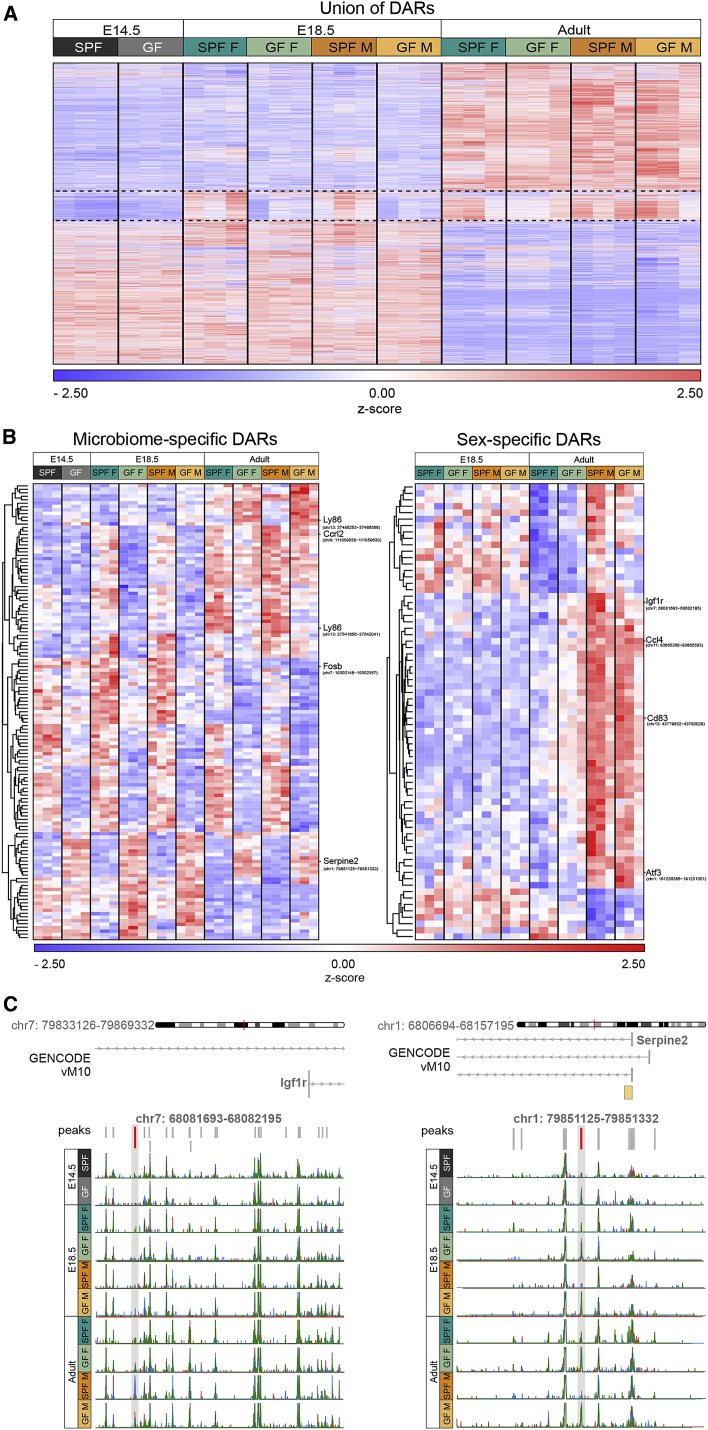
ATAC-Seq Reveals Temporal Changes in Chromatin Accessibility in the Absence of the Microbiome (A) Heatmap showing the hierarchical clustering of all DARs (FDR <0.1, #17,617) colored according to z-transformed read counts (cpm) from blue (low count) to red (high count), in microglia from SPF and GF mice. Each row is a biological replicate, with each replicate obtained by pooling microglia from 1–3 brains. (B) Heatmaps showing the hierarchical clustering of microbiome- or sex-specific DARs (FDR <0.1) colored as in (A). Each row is a biological replicate. (C) Normalized ATAC-seq read coverage of two representative loci. Displayed gene models are taken from the GENCODE vM10 annotation. The DAR of interest is highlighted in red. Blue, green, and red lines indicate the three samples from one group. See also Figure S7 and Table S4.

**Figure S7:**
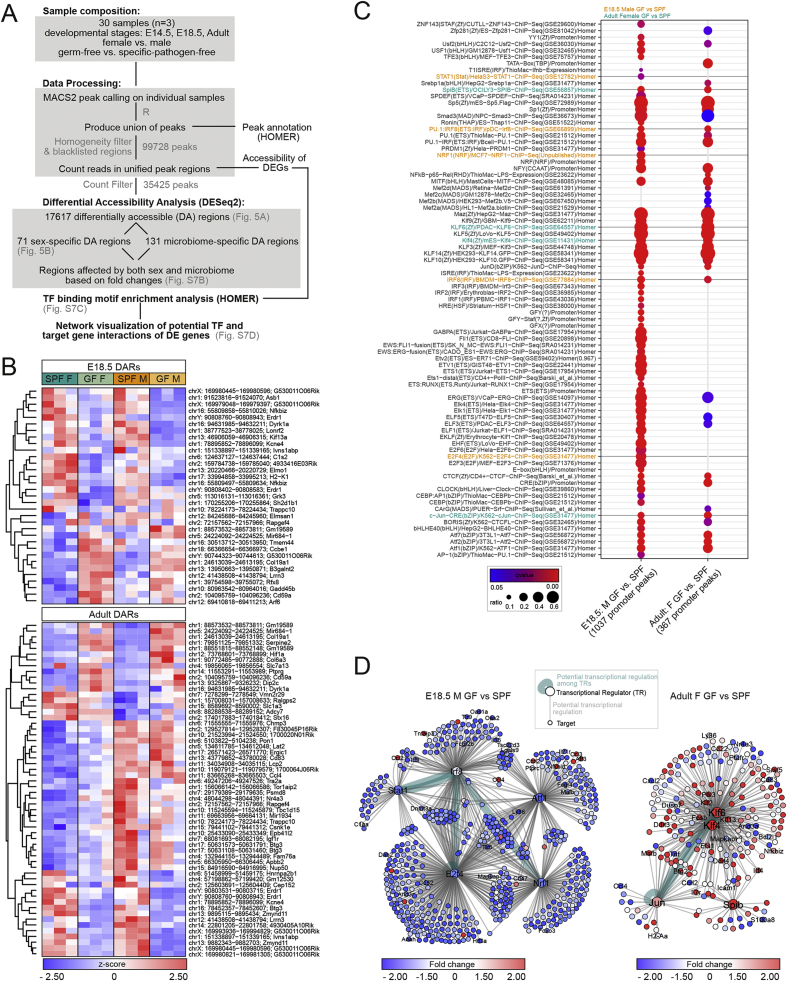
ATAC-Seq Reveals Temporal Changes in Chromatin Accessibility of Germ-Free Mice, Related to Figure 5 (A) Schema illustrating the workflow of the bioinformatics ATAC-seq analysis in microglia from SPF and GF mice at E14.5, E18.5 and in adult. (B) Heatmap showing the hierarchical clustering of the DARs (FDR < 0.1) affected by both sex and microbiome with a FC of at least 1.5 due to both factors, colored according to z-transformed read counts (cpm) from blue (low count) to red (high count) in microglia from SPF and GF mice at E18.5 and in adult. n = 3 replicates per condition and stage, with each replicate obtained by pooling microglia from 1 to 3 brains. (C) Dot plot showing significantly enriched transcription factor binding motifs (q-value < 0.05) in the ATAC-seq peak sequences found in promoter regions of the indicated sets of DEGs. Dot size indicates the ratio of sequences featuring the respective motif to the total number of tested sequences, and dot color illustrates the q-value of the enrichment. Green motifs correspond to transcription factors differentially expressed between GF and SPF male microglia at E18.5 and orange motifs to transcription factors differentially expressed between GF and SPF female adult microglia. (D) Network visualization of differentially-expressed transcription factors corresponding to enriched binding motifs and their potential target genes among the DEGs between E18.5 male GF and SPF (left panel) and adult female GF and SPF (right panel). Grey edges indicate a potential regulation of the target gene by the transcription factor and turquoise edges present potential regulation between transcription factors. Nodes are colored according to their FC of the indicated comparison. See also Tables S4 and S5.

To further assess the impact of the GF status, we performed Assay for Transposase Accessible Chromatin sequencing (ATAC-seq)(Buenrostro et al., 2013, Lara-Astiaso et al., 2014) on FACS-purified microglia from brains of E14.5 and E18.5 embryos and of P60 adults (Figures 5 and S7). By analyzing the union of differentially accessible regions (DARs) across all conditions, we first observed that the overall chromatin accessibility landscape is markedly different in embryonic versus adult microglia, in both SPF and GF conditions (Figure 5A; Table S4), consistent with previous findings (Matcovitch-Natan et al., 2016). Interestingly, from E14.5 to E18.5, the accessibility of several regions increased in embryos of SPF but not GF mice, revealing a mild but global prenatal impact of the absence of maternal microbiota (Figure 5A). We then focused on DARs that vary between GF and SPF conditions from E14.5 to adults, hereinafter referred to as microbiome-specific DARs (Figure 5B, left panel; Table S4). We observed that microbiome-specific DARs differed across stages, but noticed a larger overlap between E14.5 and E18.5 compared to adult DARs (Figure 5B, left panel). Among the genes associated with microbiome-specific DARs, we found *Ly86* and *Fosb* (Figure 5B, left panel), which are involved in microglial function and LPS responses. In parallel, we examined sex-specific DARs at E18.5 and in adults, excluding those located on the X and Y chromosomes, and found several DAR-associated genes linked to microglial activity, including *Ccl4*, *Cd83*, and *Atf3* (Figure 5B, right panel; Table S4). Interestingly, whereas adult SPF and GF male microglial chromatin accessibility landscapes appeared similar, female samples displayed more notable differences (Figure 5B, right panel), revealing a sexually biased modification, as observed in transcriptomic analyses. Consistently, the overlap between microbiome- and sex-specific DARs highlighted several sex-specific responses to the absence of microbiota in adult mice, while this dimorphism was less obvious at E18.5 (Figure S7B). Importantly, GF conditions led, in most cases, to a reduction in DARs, as illustrated with *Igf1r* (Figure 5C, left) and less frequently to the appearance of additional regions, as illustrated with *Serpine2* (Figure 5C, right). Thus, chromatin accessibility is modulated by the presence of the microbiome, starting prenatally.

These observations, however, cannot account for the differences we observed at the RNA level, as the overall accessibility of the DEGs identified by transcriptomics was mostly unaffected. We thus performed an *in silico* analysis to identify putative regulatory transcriptional networks within the DEGs. By delineating potential regulatory regions in the promoters of the DEGs using the ATAC-seq results, we searched for enriched occurrence of TF binding sites using Homer software (Figure S7A). Notably, we found that several of these TFs were differentially expressed and by screening for their putative target genes among the DEGs, we predicted networks of highly deregulated genes in either SPF versus GF E18.5 males, or in SPF versus GF adult females (Figure S7C). Such deregulated regulatory networks included TFs that have already been involved in microglial differentiation, such as *Irf8* (Kierdorf et al., 2013, Masuda et al., 2012) and *Stat1*, or that have been shown to be involved in responses to inflammation, such as *Klf2/4/6* and *Jun/Fos* (Figure S7D; Table S5).

Altogether, these data suggest that the presence of the microbiota induces subtle, but global, chromatin accessibility changes during embryonic microglial development and regulates several key targets, such as *Ly86*. In addition, we observed sex-specific responses in adults, where females appeared to be more affected by the absence of microbiota, consistent with our transcriptomic analyses.

### Antibiotic Treatment Induces Mild Sexually Biased Transcriptomic Modifications in Microglia

**Figure 6:**
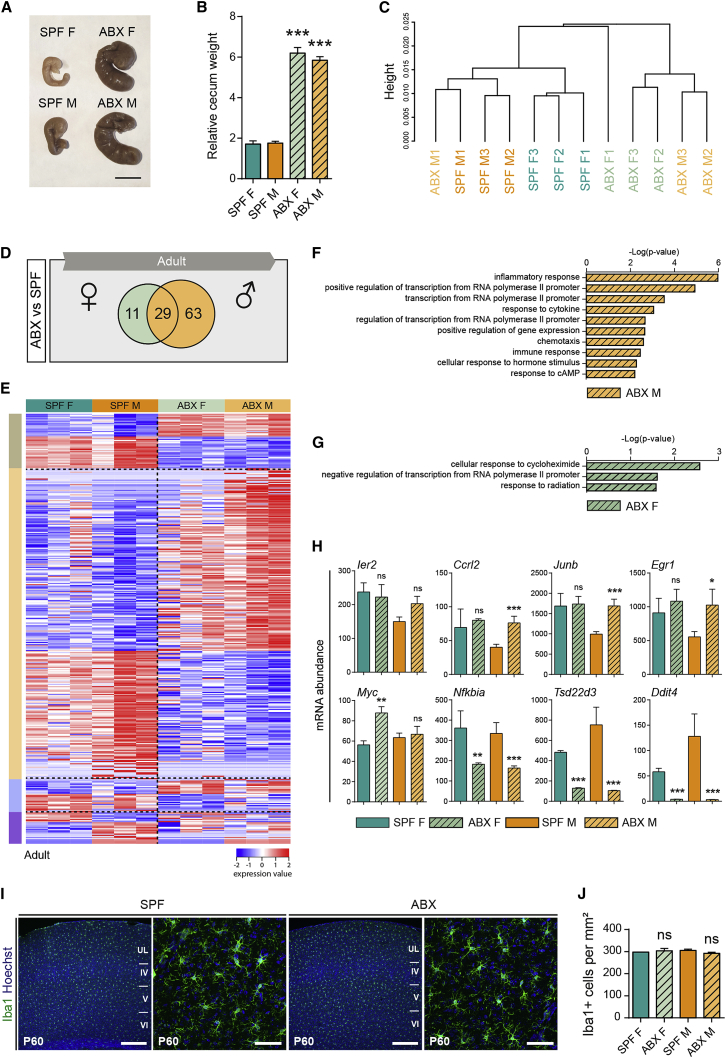
Acute Antibiotic Treatment Induces Mild Sexually Biased Transcriptomic Modifications (A and B) Representative images of cecum from control and ABX adult mice (A) and their weight (adjusted for body weight) (B). One-way ANOVA with Tukey post hoc test was used to assess differences. ^∗∗∗^p < 0.001. n = 12 mice per condition and sex. (C) Dendrogram illustrating hierarchical clustering of microglial transcriptomes from control and ABX adult mice. n = 3 biological replicates per condition and sex, each replicate containing microglia from 3 brains. (D) Number of genes showing at least a 1.5-fold difference in expression level between microglia from females and males of control and ABX mice. n = 3 biological replicates per condition and sex. (E) Heatmap of DEGs in adult microglia from ABX and SPF males. Color codes on the left highlight DEGs different across conditions (brown SPF/ABX; beige males SPF/ABX; blue females SPF/ABX; purple SPF female/male). Each row is a biological replicate, n = 3 replicates per condition and sex. (F and G) Signaling pathways analysis of the DEGs showing at least 1.5-fold lower expression level in microglia from males ABX versus male controls (F) and from females ABX versus female controls (G). (H) mRNA levels abundance for some representative DEGs from RNA-seq dataset. n = 3 per condition and sex. (I and J) Coronal sections of the somatosensory neocortex of P60 control and ABX-treated mice showing Iba1^+^ microglia (I) and quantification of their density in the cortical plate (J). Scale bars low magnification, 300 μm; scale bars high magnification, 50 μm. n = 3 mice per stage and condition. Two-sided unpaired Mann-Whitney test was performed to assess differences. ns, not significant. Data are represented as means ± SEM. See also Table S6.

The observed impact of GF status on adult microglia could result from the lifelong absence of maternal microbiota and/or from the lack of microbiome in adults (Erny et al., 2015). To address this issue, we performed RNA-seq of FACS-purified microglia from brains of adult SPF mice that had received 1 week of antibiotic treatment (ABX) and compared them to untreated SPF controls. ABX treatment induced significant increases in cecum weight and size, indicative of decreased microbiota, in both sexes (Figures 6A and 6B). Microglial transcriptomes from these mice clustered mostly by control/ABX and also by sex (Figure 6C). DEG analysis showed that ABX treatment triggered transcriptomic changes in microglia (Figures 3 and S3): 92 genes were differentially expressed between microglia from SPF and ABX males, whereas 40 genes were differentially expressed between microglia from SPF and ABX females (Figures 6D and 6E; Table S6). Among the ABX-induced DEGs, we identified a sex-specific signature of 63 DEGs in ABX males, including *Ccrl2*, *Junb*, and *Egr1* (Figure 6H), of 11 DEGs in ABX females, including as *Myc* (Figure 6H), as well as a common signature of 29 genes dysregulated in microglia from both ABX-treated males and females including *Nfkbia*, *Tsd22d3*, and *Ddit4* (Figures 6D and 6H). Thus, ABX treatment has a sexually dimorphic impact on microglial transcriptomes as well as a shared response in males and females (Figures 6D and 6E). The top DAVID signaling pathways that emerged in adult ABX males were linked to immune response, whereas microglia from female ABX adults showed more marked modulation of expression of genes associated with regulation of transcription (Figures 6F and 6G; Table S6). The common changes in microglial expression signature induced by ABX in both sexes were linked to signal transduction, modulation of transcription, and response to stress (Table S6). We further assessed microglial density or morphology and found no significant differences in either ABX males or females compared to untreated SPF controls (Figures 6I and 6J).

Taken together, our observations show that while acute microbiome perturbations have a sexually dimorphic impact on microglial transcriptomic signatures, the changes observed in microglia of adult GF mice result predominantly from the long-term developmental effect of the absence of the microbiome.

### A Core Transcriptomic Signature Exists within Human and Mouse Mid-gestation Fetal Microglia

**Figure 7:**
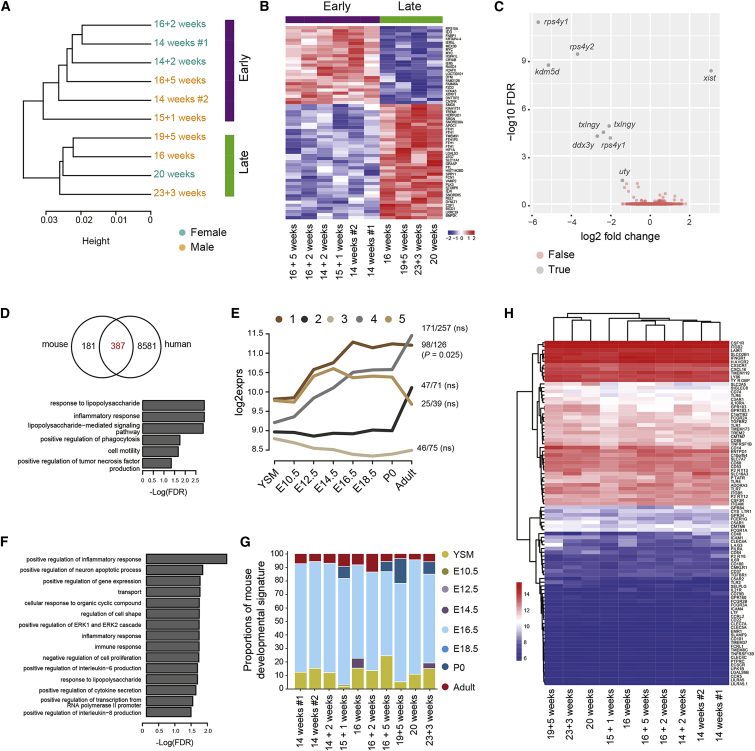
Human Mid-gestation Fetal Microglia Share Features of Murine Fetal Microglia (A) Dendrogram illustrating hierarchical clustering of human fetal microglia transcriptomes. n = 10. (B) Heatmap of DEGs between microglia from early and late mid-gestation fetal clusters. Each row is a biological replicate. (C) Volcano plot of DEGs between microglia from male and female fetuses. n = 10. (D) Venn diagram of murine microglial core genes and genes expressed in all human samples with GO enrichment of the 387 common DEGs. FDR, false discovery rate. (E) Enrichment of mouse-human common signature genes in the five clusters of mouse core signatures. (F) GO enrichment by cluster 1 of mouse core signature. FDR, false discovery rate. (G) CIBERSORT analysis of human microglia with mouse developmental signatures. (H) Heatmap of the expression of sensome genes in human fetal microglia. Each row is a biological replicate; n = 10. See also Table S7.

To start investigating how our findings in mice might relate to humans, we examined gene expression in FACS-purified microglia from human pregnancy termination male and female fetuses between 14 and 24 weeks estimated gestational age (Figure 7).

Hierarchical analysis identified two main clusters that were independent of fetal sex, instead correlating with early versus late mid-gestation and characterized by 63 DEGs (Figures 7A and 7B; Table S7). DEGs between female and male fetal microglial samples included 9 genes only present on X and Y chromosomes (Figure 7C), consistent with a lack of sexual dimorphism in mid-gestation human microglia. To investigate similarities between human and mouse microglial gene expression datasets, we first identified a core signature (568 genes) specific to mouse microglia across all developmental stages and found that 387 of their human counterparts were also expressed in all fetal samples and predominantly associated with immune response and phagocytosis (Figure 7D; Table S7). We then built clusters in these 568 genes and examined their relative enrichment at different stages of development (Figure 7E and 7F; Table S7), revealing high overall conservation in the core transcriptomic profile. We then used our mouse microglial transcriptomic signature (Figure 1) as a reference to show that mid-trimester human fetal microglia appears most closely related to the mouse E16.5 signature (Figure 7G). Finally, to explore whether human fetal microglia might have the capacity to detect environmental changes and react to them, we analyzed their expression of sensome genes. Some of the sensome genes was already highly expressed in mid-gestation (Figure 7H; Table S7), including *Csf1R*, *Cx3cr1*, *Ifngr1*, *Cxcl16*, and *Ly86*. Altogether, these results suggest that mid-gestation human fetal microglia are not sexually dimorphic, but have already acquired expression of sensome-related genes that could render them capable of responding to environmental challenges *in utero*, as observed in mice.

## Discussion

Here, we reveal that microglia, major contributors to brain circuits in normal and pathological conditions, exhibit sex- and age-specific alterations in gene expression in response to the absence of the microbiome. Prenatal microglia, which normally show limited sexual dimorphism, were severely impacted in GF males. In adults, microglia showed sexually biased responses with both acute and long-term contributions of the microbiome. Because several microglia-associated pathologies exhibit sexual biases, our findings have major implications regarding the roles of microglia in health and disease.

### Developmental Maturation of Microglia from Embryonic to Adult Stages

Consistent with a previous study (Matcovitch-Natan et al., 2016), we found that microglia undergo distinct phases of differentiation with a striking reinforcement of immune-related gene expression and difference in global chromatin accessibility landscapes between prenatal and adult microglia. These findings emphasize the dynamic maturation process of microglia, likely resulting from the integration of systemic signals and cues from the local brain environment. In contrast, embryonic phases are associated with key features of neuronal development and morphogenesis. Consistently, we found that the cytokine receptor gene *Cxcr4* regulates microglial colonization of the neocortex. Thus, our study enabled the identification of a candidate gene for stage-specific functions and highlights the need to better describe embryonic microglial functions, which just begin to be unraveled (Thion and Garel, 2017).

Importantly, the core mouse microglial gene expression signature is significantly conserved in human mid-gestation fetuses, revealing remarkable parallels between these species in microglial differentiation. Additional analyses in other species, using different purification techniques, or across brain regions, will be instrumental to define conserved pathways, as well as to identify potential species-specific divergences or spatial heterogeneity of the microglial population (Grabert et al., 2016). Nevertheless, in both murine and human microglia, multiple sensome genes were expressed prenatally, reinforcing the idea that embryonic microglia could constitute an entry point for local and systemic signals in developing brain circuits (Gosselin et al., 2014, Gosselin et al., 2017, Lavin et al., 2014).

### A Progressive Acquisition of Microglial Sexual Dimorphism

Our analyses revealed that only a few, predominantly X- and Y-linked, genes were differentially expressed between embryonic male and female microglia in both mice and humans. In adults, murine microglia from females showed increased expression of immune response-related genes than their male counterparts, consistent with a higher basal level of immune activation in females (Klein and Flanagan, 2016). For instance, genes involved in type I interferon (IFN-I) pathways, including *Ifnb1*, *Ccrl2*, and *Cxcl10*, were more highly expressed in microglia from females, which could have important implications for immunological studies. Of note, we found that the sexual dimorphism in adult microglia showed some variability across housing facilities, because the dimorphism in SPF controls of our ABX-treated mice was less pronounced than in controls of GF mice. One modulating factor, as we demonstrate in this study, is the presence of the microbiome. Indeed, the magnitude of the adult microglial sexually dimorphic signature, comprising genes involved in immune responses, was markedly reduced in GF mice, arguing that the microbiome has an influence on its normal emergence. Because microglia sex differences remain poorly described in the literature, it will be essential to explore this feature in additional conditions and species, as well as to dissect the underlying mechanisms. At this point, our findings reveal that the cellular properties of microglia that support proper brain functions might be sexually biased and warrant further investigation for a better understanding of their consequences in health and disease.

### Sex- and Time-Specific Impacts of Long-Term Absence of the Microbiome

The lack of maternal microbiota clearly influenced microglial cells in the fetal brain, at both the transcriptomic and chromatin accessibility levels. Thus, the microbiome is likely contributing to the maturation of microglial cells already *in utero*. Among the genes regulated, *Ly86* and *Aoah* are involved in the response to LPS, raising the intriguing possibility that maternal microbiota might prime microglia for their response to postnatal challenges. Several reports, for instance in intestinal lymphocytes (Semenkovich et al., 2016), reveal an importance of the microbiome in maturation, suggesting that it might be a broad tuner of the immune system.

Importantly, microglia exhibit sex-specific temporal windows of susceptibility to the long-term absence of the microbiome, with males being more affected early during *in utero* development and females showing instead profound changes in adulthood. Irrespective of the stage, microglia from GF mice displayed transcriptomic alterations in immune response genes and might be linked to a more immature or hypo-activated immune state. In adults, the baseline increase in activation state of female microglia might explain why these cells are more affected than their male counterparts by the absence of microbiota. In contrast, embryonic microglia are not markedly sexually dimorphic in the steady state, raising questions on the origin of their sexually dimorphic responses in GF embryos. Thus, independent of whether microglia are sexually dimorphic in steady-state, they show age-dependent sex-specific differences under GF conditions. This issue is crucial, because sex-specific modulation of symptoms by the microbiota has been reported in models of several diseases including Parkinson's disease or type 1 diabetes (Markle et al., 2013, Sampson et al., 2016, Yurkovetskiy et al., 2013). Irrespective of the underlying mechanisms, we identified distinct temporal windows for enhanced microglial alterations in response to long-term environmental challenges. Remarkably, epidemiological studies show that males are more susceptible to embryonic and early life challenges such as inflammation, leading to an increased risk factor for ASD or earlier onset of schizophrenia (Halladay et al., 2015, Ochoa et al., 2012). Conversely, depression, auto-immune diseases, and MS, which develop in adolescents or adults, show a higher occurrence rate in females (Ngo et al., 2014, Zagni et al., 2016). Thus, our results reveal that microglia act as time- and sex-dependent responders to environmental challenges, with a potential direct influence on the etiology of the sex bias observed in pathologies associated with microglial dysfunction.

### Short- and Long-Term Influences of the Microbiome on Adult Microglia

Acute microbiome depletion through short-term ABX treatment in adult induced a mild, rapid, and sexually dimorphic change in microglial transcriptomic signatures, revealing that the microbiome has a dynamic impact, which seems biased in males versus females. Thus, antibiotic treatment shows the potential for mild reactive changes in microglia, but also illustrates that the majority of microglial transcriptomic changes seen in GF mice are a result of long-term effects of the lack of microbiome, specifically in females. This phenomenon is reminiscent of other parts of the immune system, where microbiota are required for the normal maturation and development (Honda and Littman, 2016), albeit sexually dimorphic traits have not been largely investigated so far. Importantly, our work reveals that microglia exhibit sexually biased responses to acute or long-term microbiome depletions. This finding reinforces the view that the gut flora has wide-scale impact, which is supported by a recent study showing that differences in microbiota composition of wild and laboratory mice modifies immune responses (Rosshart et al., 2017).

Altogether, our study reveals an important surprising interplay between sex-dependent cellular features of microglia and environmental factors, in this case the microbiome, which has major implications for our understanding of normal development and disease. More generally, this study highlights the urgent necessity to systematically specify the sex of the subjects in clinical and pre-clinical studies and for this sexual dimorphism at the microglial level to be taken into account when interpreting results.

## STAR★Methods

### Key Resource Table

**Table:** 

REAGENT or RESOURCE	SOURCE	IDENTIFIER
**Antibodies**		

Rat Anti-Mouse CD45 APC-Cy7 Conjugated	BD Biosciences	Cat# 557659; RRID:AB_396774
BV650 Rat Anti-Mouse/Human CD11b	BioLegend	Cat# 101239; RRID:AB_11125575
Biotin Anti-Mouse F4/80	BioLegend	Cat# 123106; RRID:AB_893501
Rat Anti-Mouse Ly-6C PerCP-Cy5.5	eBiosciences	Cat# 45-5932-82; RRID:AB_1518762
Mouse Anti-Mouse CD64, PE Conjugated	BD Biosciences	Cat# 558455; RRID:AB_647241
Mouse Anti-Human CD45, V500 Conjugated	BD Biosciences	Cat# 560777; RRID:AB_1937324
Mouse Anti-Human CD11b, PE-Cy7	eBiosciences	Cat# 25-0118-42; RRID:AB_1582272
Mouse Anti-βIII Tubulin	Promega	Cat# G7121; RRID:AB_430874
Rat Anti-Human/Mouse CTIP2	Abcam	Cat# ab18465; RRID:AB_2064130
Rabbit Anti-Human/Mouse/Rat CUX1	Santa Cruz Biotechnology	Cat# sc13024; RRID:AB_2261231
Rat Anti-Mouse CD184 (CXCR4)	BD Biosiences	Cat# 551852; RRID:AB_394273
Rabbit Anti-Iba-1	Wako	Cat# 01919741; RRID:AB_839504
Rat Anti-Neural Cell Adhesion Molecule L1	Millipore	Cat# MAB5272; RRID:AB_2133200
Rabbit Anti-Human/Mouse TBR1	Abcam	Cat# ab31940; RRID:AB_2200219
Rabbit Anti-Mouse P2Y12	AnaSpec	Cat# 55043A; RRID:AB_2298886
Goat Anti-Mouse VEGFR2	R&D system	Cat# AF644; RRID:AB_355500
Rabbit Anti-monomeric Azami-Green 1	MBL International	Cat# PM052; RRID:AB_10597577
Fluorescein labeled GSL I isolectin B4	Vector	Cat# FL-1201; RRID:AB_2314663
AlexaFluor 488-conjugated Donkey anti-Chicken	Jackson ImmunoResearch Labs	Cat# 703-545-155; RRID:AB_2340375
AlexaFluor 488-conjugated Donkey anti-Guinea pig	Jackson ImmunoResearch Labs	Cat# 706-545-148; RRID:AB_2340472
AlexaFluor 488-conjugated Donkey anti-mouse	Jackson ImmunoResearch Labs	Cat# 715-545-150; RRID:AB_2340846
AlexaFluor 488-conjugated Donkey anti-rat	Jackson ImmunoResearch Labs	Cat# 712-545-150; RRID:AB_2340683
Cy3-conjugated Donkey anti-Goat	Jackson ImmunoResearch Labs	Cat# 705-165-003; RRID:AB_2340411
Cy3- conjugated Donkey anti-Mouse	Jackson ImmunoResearch Labs	Cat# 715-165-151; RRID:AB_2315777
Cy3-conjugated Donkey anti-Rabbit	Jackson ImmunoResearch Labs	Cat# 711-165-152; RRID:AB_2307443
Cy5-conjugated Donkey anti-Goat	Jackson ImmunoResearch Labs	Cat# 705-175-147; RRID:AB_2340415
Cy5-conjugated Donkey anti-Mouse	Jackson ImmunoResearch Labs	Cat# 715-175-151; RRID:AB_2340820
Cy5-conjugated Donkey anti-Rabbit	Jackson ImmunoResearch Labs	Cat# 711-175-152; RRID:AB_2340607
Cy5-conjugated Donkey anti-Rat	Jackson ImmunoResearch Labs	Cat# 712-175-153; RRID:AB_2340672

**Chemicals, Peptides, and Recombinant Proteins**		

Amphotericin B solubilized	Sigma-Aldrich	Cat# A9528
Ampicillin sodium salt	Sigma-Aldrich	Cat# A9518
Colistin sulfate salt	Sigma-Aldrich	Cat #C4461
Collagenase type IV	Sigma-Aldrich	Cat# C5138
Corn oil	Sigma-Aldrich	Cat# C8267
DNase I	Roche	Cat# 10104159001
Fetal Bovine Serum	GIBCO	Cat# 10270-106
Fetal Calf serum	Serena	Cat# S-FBS-SA-015
Hoechst	Sigma-Aldrich	Cat# 33342
Paraformaldehyde	Sigma-Aldrich	Cat# P6148
Percoll	GE Healthcare	Cat# 17089101
RPMI	eBioscience	Cat# 00433357
Streptomycin sulfate salt	Sigma-Aldrich	Cat #S6501
Tamoxifen	Sigma-Aldrich	Cat# T5648
Triton 100X	Eurobio	Cat# GAUTTR00-07

**Critical Commercial Assays**		

Ambion *mir*Vana miRNA Isolation Kit	Life Technologies	Cat# AM1560
RNAqueous-Micro Total RNA Isolation Kit	Thermo Fisher Scientific	Cat#AM1931
DNA High Sensitivity Reagent Kit	PerkinElmer	Cat# CLS760672
Illumina Nextera XT kit	Illumina	Cat# FC-131-1024
KAPA SYBR FAST qPCR Kit	KAPA Biosystems	Cat# KR0392
TargetAmp-Nano Labeling Kit	Epicenter	Cat# TAN07908
TargetAmp 2-Round Biotin-aRNA Amplification Kit	Epicenter	Cat# TAB2R71010

**Deposited Data**		

Murine microarray data	This paper	GEO: GSE107129
Murine ATAC-seq data	This paper	GEO: GSE107757
Murine RNA-sequencing data SPF/GF	This paper	GEO: GSE107925
Murine RNA-sequencing data SPF antibiotics	This paper	GEO: GSE108045
Human microarray data	This paper	GEO: GSE107128

**Experimental Models: Organisms/Strains**		

Mouse: FUCCI (fluorescent, ubiquitination-based cell cycle indicator)	A^∗^STAR Biological Resource Centre	Riken: RBRC02704
Mouse: C57BL/6j SPF	A^∗^STAR Biological Resource Centre	N/A
Mouse: *Sall1*^*gfp/+*^	Institute of experimental Immunology Zurich	(Takasato et al., 2004)
Mouse: B6.129P2-*Cxcr4*^*tm2Yzo*^	The Jackson Laboratory	JAX: 008767
Mouse: B6.129P2(C)-*Cx3cr1*^*tm2.1(cre/ERT2)Jung*^/J	Steffen Jung	JAX: 020940
Mouse: C57BL/6j SPF	SPF C57BL/6 were housed in Petterson lab animal facility	N/A
Mouse: C57BL/6j SPF and GF	GF C57BL/6 origin from the colony housed in Petterson lab animal facility	N/A
Mouse: C57BL/6j SPF	IBENS	N/A

**Oligonucleotides**		

RTqPCR primers	Table S3	N/A

**Software and Algorithms**		

Adobe Photoshop	N/A	http://www.adobe.com
Bioconductor GenomicRanges v1.28.4	(Lawrence et al., 2013)	http://bioconductor.org/packages/release/bioc/html/GenomicRanges.html,RRID:SCR_000025
Bioconductor GenomicAlignments v1.12.2	(Lawrence et al., 2013)	http://bioconductor.org/packages/release/bioc/html/GenomicAlignments.html
Bioconductor DESeq2 v1.16.1	(Love et al., 2014)	http://bioconductor.org/packages/release/bioc/html/DESeq2.html, RRID:SCR_015687
Bioconductor Gviz v1.20.0	(Hahne and Ivanek, 2016)	http://bioconductor.org/packages/release/bioc/html/Gviz.html
Biolayout Express 3D 3.3	(Theocharidis et al., 2009)	http://www.biolayout.org/, RRID:SCR_007179
Bowtie v1.1.1	(Langmead et al., 2009)	http://bowtie-bio.sourceforge.net/index.shtml, RRID:SCR_005476
CIBERSORT analysis	(Newman et al., 2015)	https://cibersort.stanford.edu/
Cytoscape 3.4.0 and 3.5.1	(Shannon et al., 2003)	http://www.cytoscape.org/, RRID:SCR_003032
Database for Annotation, Visualization and Integrated Discovery	(Huang et al., 2009)	https://david.ncifcrf.gov/
EdgeR package	(Robinson et al., 2010)	http://bioconductor.org/packages/release/bioc/html/edgeR.html
FeatureCount program	(Liao et al., 2014)	http://subread.sourceforge.net/
FlowJo v6.05	FlowJo	https://www.flowjo.com/
GraphPad Prism v6.05	GraphPad Software	https://www.graphpad.com/scientific-software/prism/
HOMER v4.9.1	(Heinz et al., 2010)	http://homer.ucsd.edu/homer/, RRID:SCR_010881
HomoloGene	NCBI	https://www.ncbi.nlm.nih.gov/homologene
Illumina GenomeStudio software	(Bibikova et al., 2011)	https://www.illumina.com/techniques/microarrays/array-data-analysis-experimental-design/genomestudio.html
ImageJ v1.50 g	NIH	https://imagej.nih.gov/ij/
Ingenuity Pathway Analysis	(Krämer et al., 2014)	https://www.qiagenbioinformatics.com/products/ingenuity-pathway-analysis/
MACS2 v2.1.0.20140616	(Zhang et al., 2008)	https://github.com/taoliu/MACS
Picard v1.134	http://broadinstitute.github.io/picard/	http://broadinstitute.github.io/picard/, RRID:SCR_006525
R package clusterProfiler	(Yu et al., 2012)	http://www.rdocumentation.org/packages/clusterProfiler
R package lumi	(Du et al., 2008)	https://www.bioconductor.org/packages/release/bioc/html/lumi.html
STAR aligner	(Dobin et al., 2013)	https://github.com/alexdobin/STAR
WGCNA v1.6.1 and 1.51	(Langfelder and Horvath, 2008)	https://cran.r-project.org/web/packages/WGCNA/index.html, RRID:SCR_003302

### Contact for Reagent and Resource Sharing

Further information and requests for resources and reagents should be directed to, and will fulfilled by, the Lead Contact, Florent Ginhoux (florent_ginhoux@immunol.a-star.edu.sg).

### Experimental Model and Subject Details

#### Animals

For generation of microarray data SPF mice from A^∗^STAR Biological Resource Centre (BRC) were used; FUCCI mice were also bred in-house at the BRC. Experiments using these mice were approved by the Institutional Animal Care and Use Committee of A^∗^STAR, Singapore (protocol 151071). Animals were bred, maintained and used under Singaporian regulations, following the recommendations of the local ethics committee.

C57BL/6j SPF and GF mice were maintained in sterile plastic isolators at the National Cancer Centre’s germ-free facility (NCC) housed in the SingHealth Experimental Medicine Centre, Singapore. Animals were maintained on autoclaved R36 Lactamin Chow (Lactamin, Sweden) and kept under 12-h light-dark cycle conditions.

*Sall1*^*gfp/+*^ mice mice were housed in specific-pathogen-free (SPF) conditions in the laboratory animal services center at the University of Zurich; experiments using these mice were approved by the Swiss Veterinary Office.

*Cxcr4*^*flox/flox*^ and *Cx3cr1*^*cre/ERT2*^ mice were housed at the Institut de Biologie de l’ENS, Paris, France. These mice were handled in accordance with European regulations following the recommendations of the local ethics committee.

For acute antibiotic treatment, SPF mice were given ampicillin (1mg/ml), streptomycin (5mg/ml), colistin (1mg/ml) and amphotericin (0.1mg/ml) in sterile drinking water *ad libitum* for one week, between 7 and 8 weeks of age. Sterile drinking water devoid of antibiotics was provided to control mice *ad libitum*.

Embryonic day (E) 0.5 was set as the day of vaginal plug formation on the dam, with postnatal day (P) 0 defined as the day of birth.

#### Human tissues

Anatomically-normal fetuses from elective mid-trimester pregnancy termination were kindly donated by patients at the Kandang Kerbau Women’s and Children’s Hospital and at the National University Hospitals in Singapore, following clear explanation of the study to the patients and their written informed consent. This work was approved by the local CIRB (approval 2013/837/D).

### Methods Details

#### Preparation of cell suspensions

Murine and human tissues were cut into small pieces, incubated in RPMI containing 10% fetal bovine serum and Collagenase type IV (0.2 mg/ml, working activity of 770U/mg; 1 hour for human tissues, adult mice and newborns and 30 minutes for embryonic mouse tissues) and then passed through a 19G needle to obtain a homogeneous cell suspension. In addition, adult brain cell suspensions were resuspended either in 40% isotonic Percoll and underlayed with 80% isotonic Percoll before centrifugation at 600 g for 20 minutes at room temperature except for adult ABX and associated controls that were resuspended in 30% isotonic Percoll. Cells at the interphase were collected and washed prior to sorting.

#### Mouse microarrays

Cells from several embryos or animals were pooled at each stage, independent of sex unless otherwise specified. Total RNA was extracted using the Ambion *mir*Vana miRNA Isolation Kit (Ambion Thermo Fisher Scientific, Waltham, MA, USA) according to manufacturer’s protocol. All mouse RNAs were analyzed using an Agilent Bioanalyser (Agilent, Santa Clara, CA, USA) for quality assessment. Biotinylated cRNA was prepared according to the protocol by TargetAmp-Nano Labeling Kit for Illumina Expression BeadChip (Epicenter (an Illumina company)) using 50ng of total RNA. 1500ng of cRNA were hybridized on Illumina Mouse WG-6 Version 2 chips (Illumina, San Diego, CA, USA) for 17 hours at 58°C. The arrays were then washed and stained according to Illumina Wash Protocol. The chips were scanned using BeadArray Scanner 500GX (Illumina, San Diego, CA, USA).

#### Human microarrays

Total RNA was isolated from human fetal microglia following the double extraction protocol: RNA isolation by acid guanidinium thiocyanate-phenol-chloroform extraction (TRIzol, Thermo Fisher Scientific) followed by a QIAGEN RNeasy Micro clean-up procedure, according to the manufacturer’s protocol. All RNAs were analyzed using an Agilent Bioanalyser (Agilent, Santa Clara, CA, USA) for quality assessment. Biotinylated cRNA was prepared according to the protocol by Epicenter TargetAmp 2-Round Biotin-aRNA Amplification Kit 3.0 (Epicenter (an Illumina company)) using 500 pg of total RNA. 750 ng of cRNA was hybridized on Illumina Human-HT12 Version 4 chips (Illumina, San Diego, CA, USA) for 17 hours at 58 C. The arrays were then washed and stained according to the Illumina Wash Protocol. The chips were scanned using a BeadArray Scanner 500GX (Illumina, San Diego, CA, USA).

#### RNA-sequencing

Cells from several embryos or animals of each sex were pooled at each stage. Their sexual identity was assessed by visual inspection and further confirmed through expression of *X* and *Y* specific genes. For GF and associated-SPF samples, Total RNA was extracted using the Ambion *mir*Vana miRNA Isolation Kit (Ambion Thermo Fisher Scientific, Waltham, MA, USA) according to the manufacturer’s protocol. All mouse RNAs were analyzed using an Agilent Bioanalyser (Agilent, Santa Clara, CA, USA) for quality assessment. Embryonic samples were processed using Smart-seq whereas adult samples were processed using Tru-seq. For Smart-seq, cDNA libraries were prepared from 2ng total RNA starting material and 1μl of a 1:50,000 dilution of ERCC RNA Spike in Controls (Ambion Thermo Fisher Scientific) using the Smart-seq v2 protocol (Picelli et al., 2014) with the following modifications: the addition of 20 μM TSO; and the use of 250pg cDNA with 1/5 reaction of Illumina Nextera XT kit (Illumina, San Diego, CA, USA). The length distribution of the cDNA libraries was monitored using a DNA High Sensitivity Reagent Kit on the Perkin Elmer Labchip (Perkin Elmer, Waltham, MA, USA). All samples were subjected to an indexed paired-end sequencing run of 2x51 cycles on an Illumina HiSeq 2000 system (Illumina)(16 samples/lane). For Tru-seq, cDNA libraries were prepared using 50 ng of total RNA and 2μl of a 1:2000 dilution of ERCC RNA Spike in Controls (Ambion). The fragmented mRNA samples were subjected to cDNA synthesis using Illumina TruSeq RNA sample preparation kit version 2 (Low-Throughput protocol)(Illumina, San Diego, CA, USA) according to manufacturer’s protocol, with the following modifications: the use of 13 PCR cycles; and using two additional rounds of Agencourt Ampure XP SPRI beads (Beckman Courter) to remove > 600bp double-stranded cDNAs. The length distribution of the cDNA libraries was monitored using DNA 1000 kits on the Agilent bioanalyzer. All samples were subjected to an indexed PE sequencing run of 2x51 cycles on an Illumina HiSeq 2000 (12 samples/lane).

For adult ABX-treated and associated controls samples, total RNA was extracted using the RNAqueous-Micro Total RNA Isolation Kit (Thermo Fisher Scientific, Waltham, MA, USA) according to the manufacturer's protocol. cDNA libraries, RNA sequencing library preparation, and Illumina sequencing were performed at the Ecole normale supérieure genomic core facility (Paris, France).10 ng of total RNA were amplified and converted to cDNA using SMART-Seq v4 Ultra Low Input RNA kit (Clontech). Afterward, an average of 150 pg of amplified cDNA was used to prepare the library with the Nextera XT DNA kit (Illumina). Libraries were multiplexed by 34 on 3 high-output flow cells. A 75 bp read sequencing was performed on a NextSeq 500 device (Illumina). A mean of 33 ± 2.4 million passing Illumina quality filter reads was obtained for each of the 12 adult SPF and ABX samples.

#### ATAC-seq

To profile for open chromatin, we used the Assay for Transposase Accessible Chromatin sequencing (ATAC-seq) protocol developed by Buenrostro et al. (Buenrostro et al., 2013), with some modification: cells were sorted in 400μl of MACS buffer (1x PBS, 0.5% BSA, 2mM EDTA) and pelleted by centrifugation for 15min at 500 g and 4°C using a swing rotor with low acceleration and brake settings. Cell pellets were washed once with PBS and cells were pelleted by centrifugation using the previous settings. Cell pellets were re-suspended in 25μl of lysis buffer (10mM Tris-HCl pH 7.4, 10mM NaCl, 3mM MgCl_2_, 0.1% or 0.5% Igepal CA-630) and nuclei were pelleted by centrifugation for 30min at 500 g, 4°C using a swing rotor with low acceleration and brake settings. Supernatant was discarded, and nuclei were re-suspended in 25μl reaction buffer containing 2μl of Tn5 transposase and 12.5μl of TD buffer (Nextera Sample preparation kit from Illumina). The reaction was incubated at 37°C for one hour before 5μl of clean up buffer (900mM NaCl, 300mM EDTA), 2μl of 5% SDS and 2μl of Proteinase K (NEB) were added and incubated for 30min at 40°C. Tagmented DNA was isolated using 2x SPRI beads cleanup. For library amplification, two sequential 9-cycle PCR were performed in order to enrich small tagmented DNA fragments. 2μl of indexing primers included in the Nextera Index kit and KAPA HiFi HotStart ready mix were used, then after the first PCR, the libraries were selected for small fragments (less than 600 bp) using SPRI cleanup. A second PCR was performed with the same conditions in order to obtain the final library. DNA concentration was measured with a Qubit fluorometer (Life Technologies) and library sizes were determined using TapeStation (Agilent Technologies). Libraries where sequenced on a NextSeq for an average of 10 million unique reads per sample.

#### *In vivo* proliferation assay

Microglial proliferation was investigated using the fluorescent ubiquitination-based cell-cycle indicator (FUCCI) transgenic mouse model, in which the green-emitting fluorescent protein Azami Green is fused to Geminin, a ubiquitination oscillator whose expression is regulated by cell-cycle-dependent proteolysis, resulting in the expression of fluorescence in cells in S/G2/M phases (Sakaue-Sawano et al., 2008). Frequency and intensity of Azami green expression in microglia were measured by flow cytometry.

#### Flow cytometry

Cell populations were identified using the antibodies listed in the Key Resources Table above, after gating for singlets and live cells. Cells were sorted for transcriptomic analysis using the BD-FACS ARIA, with marker expression measured on the BD-LSRII. For *Sall1*^*gfp/+*^ cell sorting, flow cytometry was performed using an LSRII Fortessa. Dead cells were excluded with the Fixable Viability Kit (Biolegend) or using Hoechst staining. Data were analyzed using FlowJo software (Treestar).

#### Tamoxifen treatment

Tamoxifen was prepared in corn oil and administered by oral gavage at 10mg/kg of body weight at E10.5.

#### Immunohistochemistry

Dissected brains were fixed in 4% paraformaldehyde at 4°C from 4 hours to overnight, depending on the developmental stage. Immunohistochemistry was performed on 80 μm (E14.5 to P0) or 40 μm (P20 to P60) thick free-floating vibratome sections, as previously described (Squarzoni et al., 2014). Sections were blocked for 2 hours with PBS containing 10% fetal bovine serum and 0.01% Triton X-100, and then incubated overnight with primary antibodies. Sections were rinsed twice in PBS containing 0.01% Triton X-100, followed by several PBS washes, before overnight incubation with secondary antibodies (1/400 in PBS). Hoechst (1/1000) was used for fluorescent nuclear counterstaining.

#### Image acquisition

To determine microglial density, images of the somatosensory neocortex, preoptic area and striatum were acquired with the Leica TCS SP5 confocal microscope. Images were acquired at 25X from E14.5 to E18.5 and at 10X at P20 for the somatosensory cortex. For the POA and striatum, all images were acquired at 25X. ImageJ and Adobe Photoshop were used for image processing.

#### RT-qPCR

cDNA was synthesized from 15 to 100 ng total RNA using random hexamers and SuperScript II Reverse Transcriptase (Life Technologies). Real-time quantitative PCR (RT-qPCR) was performed using KAPA SYBR Fast qPCR kit (KAPABiosystems). Three primer sets were tested for each gene and showed the same results. The primer sequences are listed in Table S3. Quantitative PCR was performed in a Roche LightCycler 480 with SYBR reagent in 20μl final volume per well. Each sample was measured in triplicate for all primers. Melt curve analysis and agarose gel electrophoresis were performed to verify the specificity of PCR amplicons.

### Quantification and Statistical Analysis

#### Mouse microarray data analysis

Raw intensity values of gene expression were extracted from microarray data using Illumina GenomeStudio software, and quantile-normalized using R package lumi. Normalized expression values were subsequently subject to log2 transformation. Hierarchical clustering was performed using Pearson correlation and the ward.D2 agglomeration method on log2-transformed expression values. Principal Component Analysis (PCA) was performed on the log2-transformed expression values using prcomp function in R, with parameter scale = TRUE. Genes that were significantly differentially-expressed between developmental stages were identified using ANOVA followed by post hoc Tukey test and with *Benjamini-Hochberg* adjusted p values less than 0.05. DEGs were divided into 7 distinct groups by hierarchical clustering. Biological functions significantly enriched by genes in each cluster were identified using Ingenuity Pathway Analysis (IPA). DEG analyses were all performed on log2-transformed values.

#### Co-expression network analysis (CENA)

For the generation of gene regulatory networks, the union of DEGs (814 genes) for all comparison and all present TFs (473 genes) was used. Expression values of these genes were used for co-expression network analysis (CENA) for all samples using BioLayout3D for DEG and TF network respectively. Applying a correlation cutoff of 0.85 for DEG network and 0.7 for TF network resulted in a co-expression network with 753 (DEG) and 431 (TF) nodes. The calculated gene-gene pairs, along with their Pearson correlation coefficients, were exported from BioLayout3D and imported into Cytoscape (Shannon et al., 2003) using force-directed layout for visualization. Group FC values (based on the comparison of each condition with the mean of all conditions) were mapped onto the network for each condition individually. To identify commonalities and differences between microglia at different stages based on co-expression network analysis, we colored genes based on their group FCs > 1.5 or < −1.5.

#### RNA sequencing data analysis

Raw reads were first aligned to mouse reference genome MM10 using STAR aligner. Read count per gene was then calculated using the FeatureCount program and gencode gene annotation version M9. Count per million read (CPM) values were calculated using the edgeR package. DEGs showing adjusted p values less than 0.05, fold change greater than 1.5, and a minimum of 25 average CPM were identified using edgeR package.

#### Weighted gene co-expression network analysis (WGCNA)

Weighted gene co-expression network analysis was performed on normalized expression data using the R package WGCNA v1.51 (Langfelder and Horvath, 2008) following the standard workflow. For computational efficiency, genes were filtered according to the subsequent procedure: First, for each comparison, the expression table was subsetted by all samples associated with the respective comparison and the 1000 most variably expressed genes between those samples were determined. The resulting 11 sets of 1000 most variably expressed genes were then united accounting for 2395 genes representing the relevant variance within the dataset. To obtain a signed network fulfilling the scale free topology, the soft-thresholding power parameter was set to 20. Co-expression modules were defined using a minimum module size of 30 genes and by merging modules with a module eigengene dissimilarity below 0.1 resulting in 10 modules having sizes between 48 and 499 genes. The modules were characterized by GO enrichment with a focus on biological processes using the R package clusterProfiler v3.0.5 (Yu et al., 2012). The co-expression network was generated based on the Topological Overlap Matrix requiring an edge weight of more than 0.3 to at least one other gene and excluding genes belonging to the gray module that contains those genes that do not fit in any other module. Network visualization was carried out in Cytoscape v3.4.0 applying the edge-weighted spring-embedded layout and hiding smaller clusters with less than 5 genes. In the base network nodes were colored according to module membership while in all other networks DEGs (Fold-Change > 1.5 or < −1.5 and CPM > 25) were highlighted by red (positive FC) or blue (negative FC) fill color. Transcription factors were marked by a triangular node shape.

#### ATAC-seq analysis

For ATAC-seq data analysis, single-end 61 bp short reads were aligned to the mouse genome version mm10 with Bowtie v1.1.1 (Langmead et al., 2009). Duplicate reads were flagged with Picard v1.134 prior to peak calling with MACS2 v2.1.0.20140616 (Zhang et al., 2008). Consensus peak regions across all 30 samples were generated with the ‘reduce’ function of the Bioconductor GenomicRanges v1.28.4 package and read counting per sample was performed using the ‘summarizeOverlaps’ function of the Bioconductor GenomicAlignments v1.12.2 package (Lawrence et al., 2013). Blacklisted genomic regions for mm10 defined by ENCODE as well as regions that were inconsistently present across samples of the same analysis group were excluded from the analysis. Regions were annotated using the HOMER v4.9.1 command annotatePeaks.pl with default parameters (Heinz et al., 2010). After filtering for a minimum count of 10 counts per million (cpm) in at least one sample, differentially-accessible (DA) regions were determined using the Bioconductor DESeq2 v1.16.1 package with a mean dispersion fit and classified as significant with a FDR-adjusted p value of 0.1 or lower (Love et al., 2014). To identify regions affected by both sex and microbiome, the union of all sex- and microbiome- specific DA regions was prepared and regions showing absolute fold changes of 1.5 or higher due to both factors were selected. To illustrate coverage tracks of ATAC signals we created normalized bigWig files using the HOMER v4.9.1 command *makeUCSCfile* with a specified fragment length of the exact read length and used the Bioconductor Gviz v1.20.0 package for visualization (Hahne and Ivanek, 2016). Chromatin accessibility of DEGs was inferred by comparing log-transformed mean counts (cpm) of the accessible regions annotated to the respective genes between given sample groups.

Transcription factor binding motif enrichment analysis was performed on ATAC-seq peak sequences identified in promoter regions of the respective DEGs using the HOMER v4.9.1 command findMotifsGenome.pl with a specified region size for motif finding of ± 200 bp from the peak center. Subsequently, differential expression of transcription factors corresponding to significantly-enriched binding motifs was assessed in the given comparison and the potential target genes were identified among the DEGs. The resulting relationships between transcription factors and target genes among the DEGs were visualized as networks using Cytoscape v3.4.0 featuring corresponding fold changes of their expression as node colors (Shannon et al., 2003).

#### Image analysis

Iba1-positive and P2Y12-positive cells were quantified using Cell Counter plugin from ImageJ software. Images were converted greys and inverted before counting.

For P2Y12 quantification in the somatosensory neocortex, CTIP2 and Hoechst staining were used to delineate the marginal zone, cortical plate, layer V and layer VI, intermediate zone and SVZ/VZ.

#### Gene ontology pathway analysis

Pathway analysis and biological function enrichment analysis were performed using ingenuity pathway analysis (IPA) for microarrays, or the Database for Annotation, Visualization and Integrated Discovery (DAVID). DAVID functional annotation was performed on the list of DEGs. Only pathways having a false discovery rate (FDR) or a p value of < 0.05 were represented.

#### RT-qPCR analysis

Quantitative values were obtained from the cycle number (Ct value). Quantities were determined as Q_target_ = 2^ΔCtsample^, where the ΔCt value was determined by subtracting the average Ct value of target gene from the average Ct value of the housekeeping gene (adapted from Beaume et al., 2011).

#### Human microarray data analysis

Raw intensity values of gene expression were extracted from microarray data using Illumina GenomeStudio software and quantile normalized using R package lumi. Normalized expression values were then log2 transformed. Hierarchical clustering of samples was performed using Pearson correlation and the ward. D2 agglomeration method on log2-transformed expression values. Hierarchical clustering identified two major groups: one corresponding to early stage fetal microglia and the other to late stage. Female and male microglia samples were discriminated by their differential expression level of *Ddx3y* and *Xist* genes. Genes that were significantly differentially expressed between microglia from early versus late fetuses, or female versus male fetuses, were selected using Limma and had adjusted p values less than 0.05. Human gene IDs and mouse gene IDs were converted to HomoloGene group ID (HID) based on NCBI HomoloGene. Converted human and murine microarray gene expression data were subjected to analysis, wherein murine data were used as reference gene expressions for identifying gene signatures of each developmental stage. The CIBERSORT analysis determined the proportion of each mouse gene signature that was represented in the human microglial samples. Genes that were expressed across all human samples were identified, and overlapped with core microglia signature genes derived from mouse microarray data (details given in the following section).

#### Core microglia signature gene identification

In order to define core signature genes of mouse microglia, we first identified genes that were expressed across all developmental stages from the yolk sac to adult. From our microarray data following murine microglial development, median gene expression values were identified for each sample. Genes with expression values higher than this median were considered “expressed”; within this list, we identified a subset of genes that were expressed in all developmental stages of microglia. However, some of these genes are also highly expressed in other cell types, therefore, we identified genes that were significantly upregulated in microglia compared to other immune cell types, using ImmGen data. The overlap between genes whose expression was upregulated in microglia and genes expressed across microglial development was identified as a candidate core signature of microglia. To further refine this candidate signature, we also removed putative house-keeping genes, identified as expressed in all cell types, from ImmGen data.

#### Statistical analyses

All data are presented as mean ± SEM. One-way ANOVA with Tukey post hoc test or two-way ANOVA with Sidak post hoc test were used to compare groups of data, and non-parametric two-tailed Mann-Whitney *U*-tests were used to compare two distributions. All graphs and statistical analyses were generated using GraphPad Prism software, unless otherwise stated. ^∗^p < 0.05, ^∗∗^ p < 0.01, ^∗∗∗^ p < 0.001.

### Data and Software Availability

The extensive datasets presented in Figure 1, Figure 2, Figure 3, Figure 4, Figure 5, Figure 6 and 7 as well as Figure S1, Figure S2, Figure S3 and S7 are available for mining (Table S1. Microarray Raw Data, Sensome Gene Lists, as well as DEGs and TFs “Clocks” during Development, Related to Figures 1, 2, and S1, Table S2. DEGs and Signaling Pathway Analysis in SPF and GF Microglia, Related to Figures 3, S2, and S3, Table S3. List of Primers Used for Real-Time qPCR, Related to Figures S2 and S3, Table S4. All Sex, Microbiome, and Sex- and Microbiome-Specific DARs from ATAC-Seq Data, Related to Figures 5 and S7, Table S5. TFs Networks from ATAC-Seq Data, Related to Figure S7, Table S6. DEGs and Signaling Pathway Analysis in Adult SPF and ABX Microglia, Related to Figure 6, Table S7. Early and Late Gene Expression, Core Mouse Microglial Gene Expression Signature, Common Human and Mouse Signature, the Five Clusters Gene Lists, and Sensome Gene Expression in Prenatal Human Microglia, Related to Figure 7).

## Supplemental Information

Table S1. Microarray Raw Data, Sensome Gene Lists, as well as DEGs and TFs “Clocks” during Development, Related to Figures 1, 2, and S1

Table S2. DEGs and Signaling Pathway Analysis in SPF and GF Microglia, Related to Figures 3, S2, and S3

Table S3. List of Primers Used for Real-Time qPCR, Related to Figures S2 and S3

Table S4. All Sex, Microbiome, and Sex- and Microbiome-Specific DARs from ATAC-Seq Data, Related to Figures 5 and S7

Table S5. TFs Networks from ATAC-Seq Data, Related to Figure S7

Table S6. DEGs and Signaling Pathway Analysis in Adult SPF and ABX Microglia, Related to Figure 6

Table S7. Early and Late Gene Expression, Core Mouse Microglial Gene Expression Signature, Common Human and Mouse Signature, the Five Clusters Gene Lists, and Sensome Gene Expression in Prenatal Human Microglia, Related to Figure 7
